# Loss of MAR1 Function is a Marker for Co-Selection of CRISPR-Induced Mutations in Plants

**DOI:** 10.3389/fgeed.2021.723384

**Published:** 2021-08-09

**Authors:** Jannis Rinne, Claus-Peter Witte, Marco Herde

**Affiliations:** Department of Molecular Nutrition and Biochemistry of Plants, Leibniz University Hanover, Hanover, Germany

**Keywords:** CRISPR, *Arabidopsis*, tomato, knockout marker, MAR1, phylogeny, co-selection

## Abstract

In this study, we describe the establishment of the knockout marker gene *MAR1* for selection of CRISPR/Cas9-edited *Arabidopsis* seedlings and tomato explants in tissue culture. *MAR1* encodes a transporter that is located in mitochondria and chloroplasts and is involved in iron homeostasis. It also opportunistically transports aminoglycoside antibiotics into these organelles and defects of the gene render plants insensitive to those compounds. Here, we show that mutations of *MAR1* induced by the CRISPR system confer kanamycin-resistance to *Arabidopsis* plants and tomato tissues. *MAR1* is single-copy in a variety of plant species and the corresponding proteins form a distinct phylogenetic clade allowing easy identification of *MAR1* orthologs in different plants. We demonstrate that in multiplexing approaches, where *Arabidopsis* seedlings were selected *via* a CRISPR/Cas9-induced kanamycin resistance mediated by *MAR1* mutation, a mutation in a second target gene was observed with higher frequency than in a control population only selected for the presence of the transgene. This so called co-selection has not been shown before to occur in plants. The technique can be employed to select for edited plants, which might be particularly useful if editing events are rare.

## Introduction

The CRISPR system is a powerful tool for the introduction of genomic mutations in a variety of organisms including many plant species. Originally adapted from a bacterial pathogen defense system, it is now one of the major biotechnological methods in the field of plant science with diverse applications ranging from fundamental research to crop optimization (Jaganathan et al., 2018; Knott and Doudna, 2018; Afzal et al., 2020; Zhu et al., 2020). The system employs the nuclease Cas9 (CRISPR associated 9) that is paired with a guide-RNA (gRNA) complementary to the targeted DNA sequence. The Cas9-gRNA complex introduces a double-strand break at the genomic target site which is repaired by the plant’s endogenous DNA repair system, either by non-homologues end joining (NHEJ) or homology-directed repair (HDR) (Knoll et al., 2014). As repair by NHEJ can lead to mutations in the target site, the CRISPR system is routinely used for the generation of frameshifts in genes of interest, resulting in incorrectly translated and non-functional proteins. The observed phenotypes in mutated plants are instrumental for elucidating the function of a gene (Malzahn et al., 2017).

Most applications of the CRISPR system in plants are routinely used in combination with a selection marker gene that is introduced to select transgenic plants before screening for the desired editing events. The presence of a transgene is undesired though and it subsequently has to be removed by backcrossing with the parental line (Kamthan et al., 2016). Additionally, the presence of a transgene does not guarantee that editing occurred and often laborious screening procedures have to be conducted. Consequently, many studies in recent years aimed to develop transient CRISPR protocols (He and Zhao, 2020). These transient methods often lack a selection mechanism for successfully edited cells.

To reduce the screening effort or for transient CRISPR protocols, it would be beneficial to select for the CRISPR-induced mutation itself, rather than the presence of a transgene. To select for the presence of a mutation in an endogenous plant gene, the knockout must confer a growth advantage for a plant or plant tissue when exposed to a toxic compound or some other detectable phenotype. One example is the mutation of the gene encoding the enzyme alcohol dehydrogenase (*ADH*), which catalyzes the conversion of allyl alcohol to toxic acrylaldehyde (Jacobs et al., 1988). Plants that are mutated in the *ADH* locus lack the ability to convert allyl alcohol and survive on medium containing the compound. It has also been demonstrated that allyl alcohol can be used to select *ADH* knockout plants mutated by the CRISPR system (Fauser et al., 2014; Castel et al., 2019). However, the presence of multiple genes of the *ADH* family in many species makes this approach impractical (Gottlieb, 1982; Fukuda et al., 2005). Alternatively, the CRISPR system was used to introduce a gain of function mutation in the acetolactate synthase gene (*ALS*) of tomato. Introduction of a point mutation in the gene renders plants insensitive to sulfonylurea herbicides (Danilo et al., 2019). Thus, the formation of a selectable marker requires a sense mutation rather than a random insertion or deletion, which can currently only be created by HDR or base editing (Knoll et al., 2014; Komor et al., 2016). The frequencies of HDR and base editing are generally lower and depend on other factors than the frequency of NHEJ, which may be problematic when using *ALS* as marker. In other approaches, genes that cause a visible phenotype are mutated. One frequently used target is the phytoene desaturase gene (*PDS*), which is involved in carotenoid biosynthesis. Knockout plants have an albino phenotype and are impaired in growth as chlorophyll and carotenoid-synthesis is disrupted (Norris et al., 1995). The gene is routinely used as a marker for CRISPR induced mutations in a variety of plant species (Di Fan et al., 2015; Nishitani et al., 2016; Naim et al., 2018). Mutations in the gene are identified visually, but the strong phenotype renders plants unusable for downstream applications.

We sought to identify a knockout marker gene (KOM) that is more universally applicable. Such a gene should be conserved and single-copy in many plant species, confer a selectable resistance when mutated by NHEJ and should not impair the performance of the plant.

Based on these prerequisites, one potential KOM candidate is *MAR1* (Multiple Antibiotic Resistance 1) of *Arabidopsis thaliana* encoded at the locus At5g26820. It is also known as *IREG3* (Iron-Regulated 3) or *FPN3* (Ferroportin 3). *MAR1* encodes a transporter involved in iron homeostasis that localizes to the membranes of chloroplasts and mitochondria (Kim et al., 2021). In addition to its function in iron transport mechanisms, it is also shown that a knockdown or knockout of the gene renders *Arabidopsis* plants insensitive to several aminoglycoside antibiotics like kanamycin (Aufsatz et al., 2009). It has been suggested that MAR1 transports these compounds opportunistically into the organelles, where they bind to ribosomes and inhibit protein biosynthesis leading to the death of the plant (Conte et al., 2009). Eukaryotic ribosomes are marginally affected, as the aminoglycoside antibiotics preferentially bind to the 16S RNA of prokaryotic ribosomes and only weakly to the eukaryotic ribosomal RNA (Padilla and Burgos, 2010). Phenotypical analysis of mutant plants revealed that plants lacking functional MAR1 have a growth phenotype under iron-deficiency, but appear normal with sufficient iron supply (Kim et al., 2021). MAR1 belongs to the ferroportin (FPN) family, which comprises three proteins in *Arabidopsis* (Schaaf et al., 2006). The other two proteins in this family Ferroportin 1 (FPN1) and Ferroportin 2 (FPN2), which are encoded at At2g38460 and At5g03570, share a sequence identity of 77% with each other, but only a 20% identity with MAR1 (Kim et al., 2021). Additionally, they localize to different cellular structures and not the chloroplasts or mitochondria (Morrissey et al., 2009). In summary, *MAR1* is a single-copy gene which is easily distinguishable from related genes in *Arabidopsis*, a knockout renders plants insensitive to aminoglycoside antibiotics and performance of mutated plants is only affected under iron-deficient conditions. These features make it an ideal KOM candidate.

The CRISPR system has multiplexing capabilities, i.e., more than one gene can be edited simultaneously in one cell (Le Cong et al., 2013). For several organisms, it was shown that it is possible to generate a selection marker by editing a suitable gene, which can be used to increase the probability to find mutations in a second target gene. This approach, called co-selection, has been used in *Caenorhabditis elegans* and *Drosophila melanogaster* (Arribere et al., 2014; Ge et al., 2016) as well as in human cell culture employing a KOM or a gain of function mutation (Moriarity et al., 2014; Agudelo et al., 2017). Co-selection may work because cells that become mutated in the marker will express an active CRISPR system, which can also edit another target. To our knowledge, it has not yet been tested whether CRISPR co-selection also works in plants. Co-selection might be useful especially for CRISPR systems that operate with low efficiencies.

In this study, we show that *AtMAR1* can be used as KOM in CRISPR experiments. Mutated *Arabidopsis* plants are insensitive to kanamycin and selection for mutations in *AtMAR1* increases the chance to find an editing event in a second target gene. A phylogenetic analysis revealed that MAR1 is conserved and encoded by single copy genes in many plant species. To demonstrate that the concept of *MAR1*-based KOM selection can easily be applied in other plants, we show that tomato explants can be selected by CRISPR-mediated mutation of *SlMAR1*. Thus, *MAR1*-based KOM selection not only works on seeds but also in tissue culture.

## Materials and Methods

### Cloning

An overview of all vectors and oligonucleotides used in this study is given in the Supplementary Tables 1, 2. Plasmids obtained from Addgene (https://www.addgene.org/) are displayed with their respective number.

Vectors for *Arabidopsis* transformation were constructed by GoldenGate-cloning utilizing the MoClo system (Weber et al., 2011). The gRNAs were designed using the CRISPR-P 2.0 design tool (Liu et al., 2017). DNA-sequences of the gRNAs were ordered as two complementary oligonucleotides (Merck), which were annealed and ligated with T4 ligase (NEB, M0202) into MoClo compatible gRNA shuttle vectors between the AtU6-26 promoter and the gRNA scaffold to generate an expression cassette (Streubel, unpublished). Two (H761) or four (H677) expression cassettes encoding gRNAs targeting At5g26820 and At1g30910 were cloned in Level 1 acceptor pICH47751 by BsaI cut-ligation. The final Level 2 vector was assembled by BpiI cut-ligation and contains: 1) a selectable phosphinotricin resistance cassette encoding a phosphinotricin acetyltransferase; 2) Cas9 expressed by the EC1.2-promoter for egg cell-specific expression (Wang et al., 2015); 3) gRNA expression cassettes and 4) a gene for a green fluorescent protein expressed by the seed specific At2S3 promoter (Aliaga-Franco et al., 2019). The vectors were transformed in the *Agrobacterium tumefaciens* strain AGL1 by electroporation.

Vectors for tomato transformation were based on a geminiviral expression system. The geminiviral replicons are delivered into the nucleus by Agrobacterium-mediated T-DNA transfer. They rapidly multiply by rolling circle amplification and expression levels of the CRISPR/Cas9-system are increased compared to systems with T-DNA integration (Čermák et al., 2015). We used pTC217 and removed the original gRNA expression cassette by NcoI/PmlI restriction digest and inserted an AtU6-26 promoter expressing a spectinomycin adenyltransferase as a dummy sequence. The dummy sequence can be exchanged by BsaI restriction digest for the gRNA expression cassette that is then expressed by the AtU6-26 promoter. Additionally, we inserted a multiple cloning site (P-395) between PmlI and SwaI restriction sites, which removed the original homology region (V118). The gRNA arrays were amplified from pGTR as described (Xie et al., 2015). DNA fragments of the arrays were inserted behind the AtU6-26 promoter of V118 to generate H386 and H387. The final vectors included the CRISPR/Cas9-system, a gRNA expression cassette with two gRNAs each (H386: gRNA3 and 4; Sequences: caa​cgc​aat​ggc​agc​agg​tg, ttg​ctt​ggc​tgg​gta​tat​gg; H387: gRNA1 and 2; Sequences: aga​gta​agg​aag​gcg​gac​ca, ctc​ctc​gct​tta​cca​cgc​aa) and the geminiviral sequences necessary for replication.

### *Arabidopsis* Handling and Transformation

*Arabidopsis thaliana* Col-0 plants were grown on soil (Steckmedium, Klasmann) in a Binder KBFW 720 with a 16/8 h light/dark-period (100 μmol s^−1^ m^−2^ light) and 70% humidity. After inflorescences developed, plants were transformed by floral dip (Clough and Bent, 1998) with *Agrobacterium tumefaciens* AGL1 carrying the constructs H761 or H677. After transformation, plants were watered for two more weeks and then dried to harvest seeds. The T1 seeds were vernalized for 48 h at 4°C and germinated on soil. Developing seedlings were sprayed with 200 mg L^−1^ glufosinate to identify transgenic plants. Transgenic lines that survived the glufosinate-treatment were grown further until seeds developed. T2 seeds were sterilized by shaking at 120 rpm for 15 min in 70% ethanol and dried on sterile paper. The seeds were placed on ½ MS-medium containing 50 mg L^−1^ kanamycin.

### Tomato Handling and Transformation

Tomato transformation was carried out in sterile *in-vitro* conditions with the cultivar MicroTom. Briefly, seeds were sterilized by shaking at 120 rpm for 20 min in a 10% hypochlorite solution. Seeds were washed in bidestilled water and germinated on ½ MS-medium containing 10 g L^−1^ sucrose under the same conditions as *Arabidopsis* plants. Cotyledons were cut from eight to ten-day-old seedlings and after removing the abaxial tip, placed on co-cultivation medium (4.3 g L^−1^ MS-salts, 112 mg L^−1^ Gamborg B5 vitamins, 30 g L^−1^ sucrose, 0.5 g L^−1^ MES, 8 g L^−1^ Phytoagar, 1 mg L^−1^ NAA, 1 mg L^−1^ BAP, and pH 5.8) for 3 days in the dark prior to transformation. In parallel, *Agrobacterium tumefaciens* AGL1 carrying H386 or H387 liquid cultures were grown with 180 rpm shaking at 28 °C overnight in 50 ml YEB-medium (5 g L^−1^ beef extract, 1 g L^−1^ yeast extract, 5 g L^−1^ peptone, 5 g L^−1^ sucrose, 10 mM MgSO_4_, and pH 7) with antibiotics as indicated. The next day, the cells were centrifuged at 4000xg for 10 min, washed and resuspended in 50 ml 10 mM MgSO_4_ with 150 µM acetosyringone. The tomato explants were incubated in the bacteria solution for 5 minutes and placed back on the co-cultivation medium. After three more days in the dark, the explants were washed with bi-distilled water containing 250 mg L^−1^ timentin and placed on shoot-inducing medium (4.3 g L^−1^ MS-salts, 112 mg L^−1^ Gamborg B5 vitamins, 10 g L^−1^ sucrose, 0.5 g L^−1^ MES, 8 g L^−1^ Phytoagar, 250 mg L^−1^ timentin, 3 mg L^−1^ zeatin riboside, and pH 5.8) with 50 mg L^−1^ kanamycin. The plates were then placed back in the light as described previously. Explants were transferred to fresh plates every 2 weeks until shoots developed.

### Detection of Editing Events

Genomic DNA was extracted from leaf material of *Arabidopsis* seedlings (Edwards et al., 1991) or tomato shoots emerging from callus (Oberacker et al., 2019). Detection of editing events was performed by amplified fragment length polymorphism analysis (AFLP) with an ABI 310 capillary sequencer (Samarut et al., 2016) or direct Sanger-sequencing of PCR amplicons (Microsynth-Seqlab). For AFLP, we amplified DNA fragments of the target locus by PCR using genomic DNA of wild type and potentially edited plants and compared the size of the amplicons to identify mutations. PCRs were set up with three oligonucleotides and performed in a 2-step protocol (95°C, 3 min; first step: [95°C, 15 s; 60°C, 15 s; 72°C, 1 min; 25 cycles]; second step: [95°C, 15 s; 52°C, 15 s; 72°C, 1 min; eight cycles]; 72°C, 3 min). Each reaction contained one forward primer with a −21 M13 overhang (gta​aaa​cga​cgg​cca​gt), a fluorescence-labelled −21 M13 forward primer 17mer (Sigma; P 2973) and a reverse primer (Schuelke, 2000). During the first step of the PCR, only the forward primer with the M13 overhang and the reverse primer bind to the DNA and generate primary amplicons. When the annealing temperature is lowered during the second step, the fluorescence labelled primers can bind to the primary amplicons and generate fluorescing DNA fragments that are detected in the capillary sequencer. Reactions with wild type genomic DNA were performed with 4,5-Dichloro-dimethoxy-fluorescein (JOE) labelled and reactions with potentially mutated DNA with 6-Carboxyfluorescein (6-FAM) labelled oligonucleotides. Samples for capillary electrophoresis were prepared by mixing 1 µl of the JOE and 6-FAM amplicons with 10 µl Hi-Di Formamide (ThermoFisher) and 0.2 µl of Orange 500 DNA Size Standard (NimaGen), incubated at 95°C for 5 min and cooled on ice. The following parameters were used for analysis of samples with the ABI 310 capillary sequencer: capillary length, 36 cm; polymer, POP-7; dye set, 5 dyes; run voltage, 15 kV; injection voltage, 15 kV; run time, 23 min; run temperature, 60°C; injection time, 5 s. Results were analyzed with GeneMapperID v3.2 (Life Technologies) and a chromatogram displayed. Peaks of different sizes for JOE and 6-FAM labelled DNA amplicons indicate the presence of insertions or deletions induced by the CRISPR system. Example chromatograms for different editing events are displayed in Supplementary Figure 1. For some samples the editing events were confirmed by Sanger sequencing. Types of insertions and deletions were identified by analyzing Sanger sequencing chromatograms using CRISPR-ID v 1.1 (Dehairs et al., 2016) and manual annotation.

### Phylogenetic Analysis

Sequences for the multiple sequence alignment and construction of the phylogenetic tree were recovered by BLASTp search on the Phytozome 12 web server using the protein sequence of *At*MAR1 as a query. Multiple alignments were performed with MUSCLE on the European Bioinformatics Institute website using default parameters. Alignments were shaded for identical or similar amino acids with the program BOXSHADE at the Expasy Bioinformatics Resource Portal. For construction of the phylogenetic tree, sequences were truncated at both termini between the protein motifs “LYASCL” and “EQRRLF” of *At*MAR1 to exclude residues with a low sequence coverage. The tree was calculated using the Maximum Likelihood method and JTT matrix-based model (Jones et al., 1992) which was previously identified as the best-fitting model. A discrete Gamma distribution was used to model evolutionary rate differences among sites (5 categories (+*G*, parameter = 0.9064)). Branch lengths of the tree indicate the number of amino acid substitutions per site. The analysis was conducted with a total of 41 amino acid sequences. The partial deletion option was employed to eliminate all positions with less than 95% site coverage from the alignment. The final dataset had 427 positions in total.

## Results

### CRISPR-Induced Mutations in At*MAR1* Render *Arabidopsis* Seedlings Kanamycin Insensitive

**FIGURE 1:**
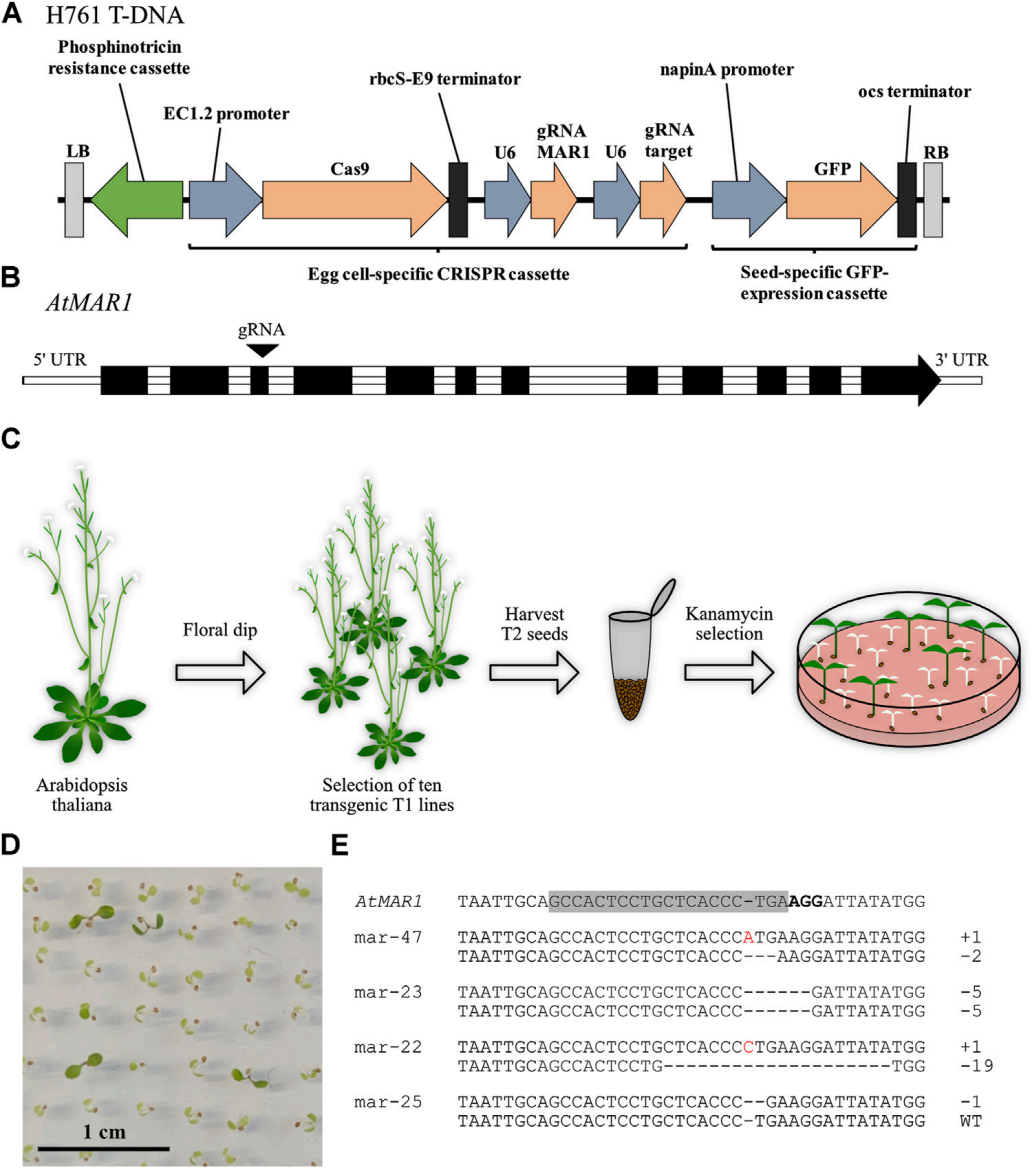
Mutations in MAR1 induced by the CRISPR system confer kanamycin resistance to *Arabidopsis thaliana* seedlings. **(A)** T-DNA of H761 that was transferred by floral dip. LB/RB: left border/right border, EC1.2: egg-cell specific 1.2, rbcS-E9: ribulose-1,5-bisphosphate carboxylase E9, U6: ubiquitin 6, GFP: green fluorescent protein, ocs: octopine synthase. **(B)** Intron-exon structure of the locus At5g26820. The gRNA target site in the third exon is indicated with an arrow. **(C)** Schematic illustration of the transformation procedure. **(D)** T2 seedlings of plants transformed with H761 selected for 14 days on ½ MS medium containing 50 mg L^−1^ kanamycin. **(E)** Examples of mutations detected in kanamycin insensitive seedlings. The area around the gRNA target site in *MAR1* is displayed with the target site highlighted in grey and the PAM in bold letters. Deleted nucleotides are indicated by dashes, inserted nucleotides are displayed in red.

To evaluate the suitability of *MAR1* as a KOM, we transformed *Arabidopsis thaliana* plants via floral dip with H761, a construct expressing the CRISPR/Cas9-system, a gRNA complementary to *AtMAR1* and a phosphinothricin acetyltransferase (Figure 1A). The target site of the gRNA is located in the third exon of *AtMAR1* (Figure 1B). We selected ten transgenic T1 lines by glufosinate treatment and analyzed them for mutations in *MAR1*. All lines only carried wild type alleles of *MAR1* and we harvested their respective T2 seeds. The seeds were germinated on ½ MS medium containing 50 mg L^−1^ kanamycin to select seedlings that were mutated in *MAR1* (Figure 1C). 14 days after germination, most seedlings started to become white and stopped growing, indicating they were still susceptible to kanamycin and thus, not mutated in *MAR1*. Other seedlings showed a kanamycin insensitive phenotype and stayed green (Figure 1D). *Arabidopsis* Col-0 plants grown in parallel on the same plates did not survive the treatment. We extracted genomic DNA from 48 green seedlings and analyzed *MAR1* for mutations by AFLP using capillary gel electrophoresis. 33 of 48 plants (68.6%) carried a homozygous or biallelic mutation in *MAR1* and 13 plants (27.1%) had heterozygous mutations. In two plants *MAR1* was wild type—these had apparently escaped the selection. We also analyzed 100 randomly chosen plants, ten of each transgenic line, for *MAR1*-editing without selecting on kanamycin to determine the general efficiency of *MAR1*-editing in our transgenic lines. The plants were grown on soil and selected by glufosinate-application to eliminate plants that lost the T-DNA by segregation. We detected *MAR1*-mutations in six plants (6%). Compared to the *MAR1*-mutation rate of 95.8% in seedlings selected on kanamycin, this shows that CRISPR-induced mutations in *MAR1* render *Arabidopsis* plants kanamycin-insensitive and that *MAR1* can be utilized as a KOM in *Arabidopsis*. The predominant type of mutation in the *MAR1* locus was a one base pair insertion, which was present in 57 of 87 alleles carrying mutations. Larger deletions of more than ten base pairs occurred in six alleles. Interestingly, only three types of larger deletions were detected (−13, −19 and −33 base pairs), each occurring twice. This indicates that there is a sequence bias which preferentially induces these kinds of larger deletions. All other detected mutations were smaller insertions between two and ten base pairs or deletions between one and ten base pairs (Supplementary Table 3). Some mutated plants were further analyzed by Sanger sequencing to determine the nucleotides that were deleted or inserted. Some examples of the determined insertions and deletions are displayed (Figure 1E). Interestingly, not all of the mutations were of homozygous or biallelic nature. We found that 13 of the 48 plants only carried a mutation in one allele, indicating that the gene dose influences the resistance or that mutated forms of MAR1 are partially dominant, negatively affecting the function of the wild type protein. In any case, the data show that a resistance can be achieved by the mutation of only one allele. Overall, it was possible to show that mutations in *MAR1* result in a selectable advantage, thus making it a promising KOM candidate.

### Co-Selection Enhances Mutation Rates in a Second Target Gene

In addition to the gRNA complementary to *MAR1*, another gRNA had been included on H761 that is complementary to a second target gene of unknown function which is encoded at At1g30910. We wanted to evaluate whether preselection of T2 seeds on kanamycin would facilitate the screening for mutations in the target gene. If the concept of co-selection was also applicable to plants, an enhanced frequency of the mutated target gene should be observed, when compared to a control group that was not selected for *MAR1* mutation. We analyzed the same 48 plants that we identified as kanamycin insensitive for mutations in the target gene and found editing events in six plants. The control group comprised the 100 plants that were grown on soil and selected with glufosinate. Here, we found a total of four mutations in the target gene. Expressed as a percentage, the target gene mutation rate of *MAR1*-selected plants was 12.5%, compared to 4% of glufosinate-selected plants (*p* < 0.1, Chi-squared test). The observed editing rate in the target gene was thus increased approximately 3-fold by preselection on kanamycin (Table 1).

**TABLE 1:** Editing frequencies of the target gene in *Arabidopsis* seedlings transformed with H761 or H677 subjected to kanamycin or glufosinate selection (Chi-squared test: *p < 0.1, **p < 0.002).

Selection method	H761			H677		
	Analyzed loci	Mutations detected	Mutation rate (%)	Analyzed loci	Mutations detected	Mutation rate (%)
Kanamycin	48	6	12.5*	128	32	25**
Glufosinate	100	4	4*	128	13	10.2**

To obtain an additional independent dataset, we analyzed target gene editing in T2 plants of a second *Arabidopsis* population, which was transformed with H677. The vector expresses two gRNAs against *MAR1* and two gRNAs against the same target gene as used above. We analyzed the two target sites of the target gene in 64 plants recovered from kanamycin selection and in the same number of plants obtained by glufosinate selection. As we had two target sites, a total of 128 respective sites were analyzed for both groups. Mutations were found in 13 of 128 sites (10.2%) in the control group and in 32 of 128 sites (25%) in the seedlings selected on kanamycin (*p* < 0.002, Chi-squared test). Thus, the discovery rate of mutations in the target gene was 2.5-fold higher after kanamycin selection (Table 1). In contrast to the variety of different mutations we found in *MAR1*, we almost exclusively detected one base pair insertions or deletions in the target gene (Supplementary Table 3).

These results show that a correlation between CRISPR-induced mutations in two unrelated loci that are targeted simultaneously can be observed in plants. Therefore, co-selection can also be used in plants as an approach to enrich for individuals containing a desired target gene editing.

### Phylogenetic Analysis Reveals that Identification of *MAR1* Orthologs in Other Plant Species is Straightforward

Because *MAR1* from *Arabidopsis* can serve as a KOM, we also wanted to assess whether potential orthologs of *MAR1* from other plant species can be used similarly. First, we conducted a phylogenetic analysis including 56 different species of vascular plants. The aim of this analysis was to find out whether orthologs of *AtMAR1* can be easily identified and are single copy genes in other plants. We conducted a BLASTp search in the Phytozome 12.1 web-based plant protein database using AtMAR1 as query, screening all available proteomes. We recovered a total of 168 protein sequences from 56 different plant species. A clear distinction between sequences with a high and low similarity towards AtMAR1 was usually observed. One or few protein sequences per organism had a higher sequence identity than 50%, while the next highest ranked sequences had identities of around 20%. We considered all sequences with a sequence identity above 50% to be likely orthologs of MAR1 from *Arabidopsis*. Sequences with a lower identity are likely orthologs of the other two members of the FPN family. In the following we will refer to orthologs with a high sequence identity as putative *MAR1* orthologs and to sequences with a lower identity as putative *FPN* orthologs. In 37 of the 56 plant species only one sequence with an identity of more than 50% was found, indicating that *MAR1* is often a single copy gene. The other 19 species had two or more similar genes, likely paralogs that originated from gene duplication events. Interestingly, all analyzed Poaceae and Panicoideae had multiple copies of putative *MAR1*, indicating an early gene duplication event in their ancestry. We provide a full table of all identified orthologs in the supplementary data (Supplementary Table 4).

**FIGURE 2:**
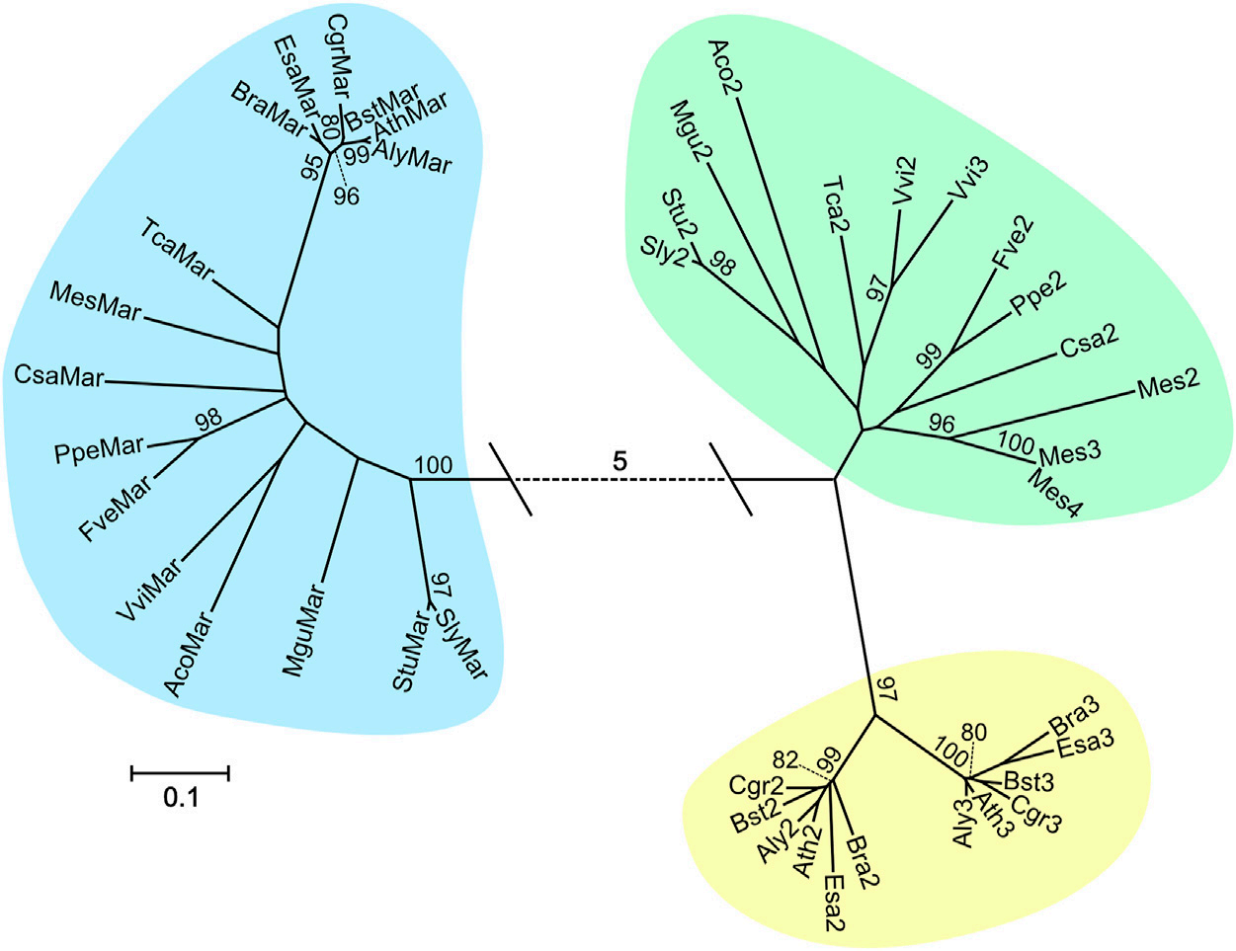
Maximum Likelihood tree constructed with sequences similar to AtMAR1 from 16 vascular plant species. The tree with the highest log likelihood (−10803.58) is displayed. 1,000 bootstrap calculations were performed and only values over 80% are shown. Branch lengths are measured in the number of amino acid substitutions per site (see legend). The dotted line represents a distance of five substitutions per site. Species names and loci are given in Supplementary Table 4, the corresponding multiple alignment is shown in Supplementary Figure 2.

We used the sequences of 16 plants with a single copy of *MAR1* to perform a multiple sequence alignment (Supplementary Figure 2) and calculate a phylogenetic tree (Figure 2). In the tree, three clades can be discerned. The clade marked with blue shading comprises all sequences identified as likely AtMAR1 orthologs and is separated by more than five amino acid substitutions per site from the two other clades. MAR1 can be readily distinguished from similar proteins in many plants, which is a prerequisite for a straightforward transfer of the *MAR1*-KOM technology established in *Arabidopsis* to other plants. The second clade (yellow shading) comprises proteins encoded by putative *FPN1* and *FPN2* orthologs from different Brassicaceae species, indicating that these proteins represent a conserved group only present in the Brassicaceae family. Sub-clades for FPN1 and FPN2, respectively, are also distinguishable. All other sequences were pooled in the last clade (green shading), which includes proteins encoded by putative *FPN* orthologs of the other analyzed species not belonging to the Brassicaceae family.

### Knockout of *MAR1* in Tomato Confers Resistance to Explants in Tissue Culture

To experimentally test the sequence-based identification of *MAR1* orthologs from the phylogenetic analysis and to evaluate whether a *MAR1*-KOM selection can be used in other species, we chose to mutate the putative *MAR1* gene in tomato (*SlMAR1*). In *Arabidopsis*, we showed that seeds uniformly mutated in *MAR1* germinate and can establish a plant in the presence of kanamycin. However, as many other plant species, tomato is routinely transformed by tissue culture techniques. In tissue culture, only a few cells will acquire the resistance and selection must work in favor of only these cells, which is different from the situation in seeds. Thus, our experiments in tomato also assess the possibility to use the *MAR1*-KOM under tissue culture conditions. *Sl*MAR1 is quite distant from *At*MAR1 in the phylogenetic analysis (Figure 2)—if *SlMAR1* also works as KOM, one can assume that less distant *MAR1* orthologs from other plants are likely to do so as well.

**FIGURE 3:**
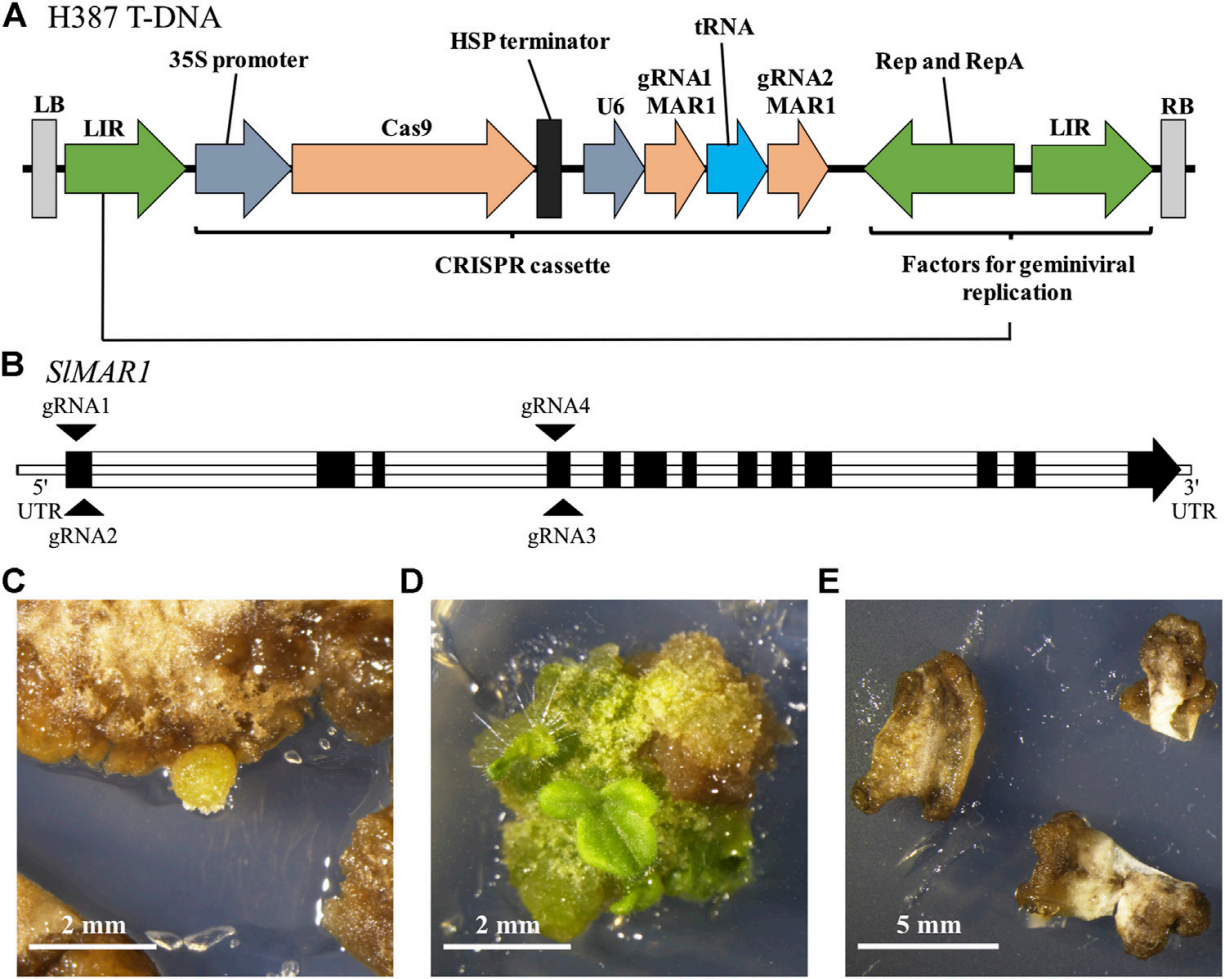
Mutations in MAR1 of tomato confer kanamycin resistance to leaf explants in tissue culture. **(A)** T-DNA of H387 that was transferred into tomato explants. LIR: long intergenic region, HSP: heat shock protein 18.2. **(B)** Intron-exon structure of the locus Solyc01g100610.3.1 encoding *Sl*MAR1. Target sites for gRNAs located in the first and fourth exon are indicated with arrows. The gRNA1 and 2 were expressed by H387 and the gRNA3 and 4 were expressed by H386. **(C)** Leaf explants were transformed and placed on shoot-inducing medium containing 50 mg L^−1^ kanamycin. Callus development occurred about 8 weeks after transformation. **(D)** Shoots developed 12 weeks after transformation. **(E)** Explants transformed with the control V118 showed no regeneration after 12 weeks.

The transformation of tomato was carried out with cotyledons as explants in sterile *in-vitro* tissue culture. We used the two constructs H386 and H387 which are based on geminiviral replicons to express the CRISPR/Cas9 system and two gRNAs complementary to *SlMAR1*, respectively, but no selection marker cassette (Figure 3A). The target sites of gRNA1 and 2 are located in the first exon and the target sites of gRNA3 and 4 in the fourth exon of *SlMAR1* (Figure 3B). We transformed 97 and 104 tomato explants with each of the two constructs and placed them on shoot inducing medium containing 50 mg L^−1^ kanamycin. As a control, 40 explants were transformed with the empty vector V118 which is not expressing the gRNAs. After 8 weeks, we observed callus development on 42 of the 97 explants transformed with H386 and 25 of the 104 explants transformed with H387. Twelve weeks after transformation, shoots emerged from eight and four calluses, respectively. The negative control showed no regeneration (Figures 3C–E). To analyze *SlMAR1* for mutations, we extracted genomic DNA from whole shoots. In total seven shoots were analyzed and we detected five biallelic and two chimeric mutation patterns. The mutations either result in a frameshift or delete a large area of the gene. In one case we found a deletion of 112 base pairs between the likely cutting sites of gRNA 1 and 2, indicating that the CRISPR/Cas9 system induced a DNA double strand break on both sites at the same time and the sequence in between was lost in the process. Overall, it is unlikely that any of the mutated *MAR1* alleles encodes a functional version of the protein (Supplementary Figure 3). These results show 1) that the sequence analysis correctly predicted the *AtMAR1* ortholog in tomato, 2) that *MAR1* of tomato can also be used as KOM and 3) that *MAR1*-KOM selection is applicable in tissue culture.

## Discussion

Selection markers play a crucial role in the identification of transgenic plants or plant tissues. As transformation rates are low in most cases, it is necessary to have a fast way of distinguishing transgenic from wild type material. In CRISPR experiments, identification of transgenic plants or plant tissues is only the first step. Transgenic material has to be screened for CRISPR-induced mutations and once they are identified, the presence of the transgene is often undesired. We demonstrate that mutation of *MAR1* can replace the transfer of a selection marker gene by directly selecting for induced mutations in the locus. There are other established selection mechanisms for CRISPR-induced mutations that do not rely on transfer of a marker gene, like the mutation of *ADH* and *PDS* or gain of function mutation in *ALS*, but each of these systems also has their downsides.

Using *ADH* is quite similar to using *MAR1* as a KOM. In both systems, a mutation induced by NHEJ is sufficient to gain a selectable growth advantage over wild type material, but in comparison to *MAR1,* the identification of *ADH* orthologs in other plant species is quite challenging. The gene is usually well annotated for model species, but for several non-model species the identification of the correct ortholog is not straightforward due to the presence of multiple isoforms and similar genes (Gottlieb, 1982; Fukuda et al., 2005). *MAR1* orthologs on the other hand are often single copy genes and their phylogenetic distance to similar genes make identification straightforward. Additionally, many tissue culture protocols rely on selection for a transgene conferring kanamycin resistance and conditions might be easily adapted to *MAR1*-KOM selection, which also confers kanamycin resistance.

*ALS* or *PDS* are also well-established markers that are used for the identification of CRISPR-induced mutations, but their mode of action differs from *MAR1* or *ADH*. Mutating *ALS* for a gain of function requires repair via HDR, which generally has a lower efficiency than gene knockout via NHEJ and requires a repair template. These additional prerequisites make its use as a marker more difficult. Additionally, attempts to directly select for HDR events in the *ALS* locus of tomato were unsuccessful and a preselection for a transgene was mandatory (Danilo et al., 2019). The authors suspect that the short timeframe was insufficient for expression of the CRISPR system and generation of the HDR event. The geminiviral expression system, utilized in this study, may result in a stronger expression of Cas9 and gRNAs allowing selection of a marker based on a HDR event.

*PDS* as a marker relies on a different selection method than *MAR1*. Mutation of *PDS* does not confer a selectable resistance, instead mutated plants are recognizable due to their albino phenotype, greatly hindering their performance. Therefore, plants carrying mutations cannot be propagated and are unsuitable for further experiments. Mutation of *MAR1* from *Arabidopsis* only causes a phenotype under iron-deficient conditions, which can be avoided by sufficient iron supply (Kim et al., 2021). It is unclear however, if loss of *MAR1* function causes other molecular phenotypes in *Arabidopsis* that influence the performance of the plant under different environmental conditions. Additionally, it has not been assessed whether loss of *MAR1* function causes stronger phenotypes in other plant species than *Arabidopsis*, especially in crop plants in an agronomical context. However, it is inherent to all systems utilizing a KOM that regenerated plants carry a non-functional gene influencing performance or physiological processes. Depending on the design and aim of an experiment, one selection mechanism may be more suitable than another, but *MAR1* represents a valuable addition to the toolkit when designing experiments aiming to select CRISPR-induced mutations.

As no transfer of a marker gene is necessary, KOMs have high potential in transient CRISPR approaches, which aim to generate targeted mutations without integrating foreign DNA into the host genome. However, they often lack a selectable marker for successfully edited material. A KOM like *MAR1* could be very valuable for that purpose as no unwanted DNA has to be crossed out and plants generated in such way are not considered transgenic in many parts of the world (He and Zhao 2020). One method for transient delivery of the CRISPR system utilizes preassembled Cas9 ribonucleoproteins that are transfected into plant protoplasts or delivered biolistically into plant tissue (Svitashev et al., 2016; Kim et al., 2017; Andersson et al., 2018). These methods lack a tool to select for induced mutations and screening procedures are often laborious (He and Zhao, 2020). Instead of conducting these screens, potentially transformed material could be screened for *MAR1* editing by application of selective pressure. That a KOM can be used for such a purpose was shown in rice blast fungus. Using ribonucleoproteins, it was possible to select for a gain of function mutation in the succinate dehydrogenase gene generating a resistance to the fungicide carboxin. Additionally, when simultaneously delivering ribonucleoproteins equipped with two different gRNAs, mutations in a second target gene were enriched. This shows that co-selection can also work in a system that employs ribonucleoproteins for editing (Foster et al., 2018). But not only the development of transient CRISPR techniques benefits from using a KOM. Studies aiming to optimize CRISPR systems in general could use *MAR1* as a screening tool as it was already demonstrated with *ADH* (Fauser et al., 2014; Castel et al., 2019).

Along with the description of *MAR1* as a KOM, we present the first demonstration of co-selection in plants. To our knowledge, co-selection facilitated by a KOM has so far only been shown in other organisms like *Caenorhabditis elegans*, *Drosophila melanogaster*, human cell culture or rice blast fungus (Arribere et al., 2014; Moriarity et al., 2014; Ge et al., 2016; Agudelo et al., 2017; Foster et al., 2018). In line with these studies, we were able to show that CRISPR-induced editing events in one locus increase the chance to also observe editing in a second, unrelated locus. A possible explanation for this linkage is that a higher expression of the CRISPR/Cas9 system results in a higher amount of induced mutations. Therefore, when selecting explants for mutations in a KOM, these explants are more likely to highly express the CRISPR/Cas9 system and consequently are also more likely to be mutated in a second target locus. In CRISPR systems that operate with low efficiencies, co-selection could be used to increase the chance of finding editing events and greatly reduce screening efforts.

In addition to the proof of concept in *Arabidopsis*, we showed that the system can also be used for the selection of mutated tomato cells in tissue culture. *In-vitro* techniques play a crucial role for the transformation of many plant species and in most species transgenic material can exclusively be generated in tissue culture. Consequently, it was important to show that *MAR1* as a KOM can also be used here. A downside of tissue culture techniques is, that regenerated plants often carry chimeric mutations induced by the CRISPR system. Although we included two gRNAs on the T-DNA, five of the seven genotyped shoots were non-chimeric. In other studies that used a selection marker for selection of T0 transformants and only one gRNA per construct, chimeric mutation patterns appeared in approximately half of the regenerated tomato plants (Yu et al., 2017; D'Ambrosio et al., 2018). Chimeric mutation patterns can be observed when CRISPR-induced mutations occur after a transgenic cell has undergone cell division. The system induces independent mutations in the divided cells and a tissue with a mix of mutations develops. When selecting *via* KOM, mutations are theoretically already induced in the first cell, as it can only proliferate after being mutated. This probably reduces the amount of chimeric plants that are generated.

Nevertheless, the system also has its limitations. We attempted to demonstrate co-selection in tomato by comparing the mutation frequency of a target gene with *MAR1* as a KOM to a geminiviral expression system with the kanamycin resistance gene neomycin phosphotransferase II. We found that the geminiviral system was so efficient in mutating the target that almost all regenerated shoots with both systems carried mutations in the target (data not shown). Consequently, tomato is either not a suitable organism to show the strength of a KOM-based approach or its benefits can only be demonstrated with target genes that are challenging to edit. It has previously been demonstrated that mutation efficiencies for different tomato genes vary and some genes are more difficult to mutate than others (Jacobs et al., 2017). We envision however, that the *MAR1*-KOM approach is especially powerful in experimental strategies that are either aiming to avoid stable integration of DNA in the plant genome (e.g., using ribonucleoprotein) or suffer from a low efficiency of DNA integration (e.g., particle bombardment). Here, our experimental scheme would enable the identification of cells that were affected by the CRISPR/Cas9 system without requiring a marker gene that is integrated into the genome. This initial selection of cells combined with a potentially increased mutation frequency especially for challenging targets may provide a significant improvement for certain experimental strategies in plant genome editing. However, the *MAR1*-KOM approach needs to be further tested in this context, involving also relevant crop plant species. Thus, our experiment in tomato is only a first step that provides proof of concept for the applicability of the *MAR1*-KOM selection in a plant species other than *Arabidopsis*, as well as in tissue culture.

We also conducted a phylogenetic analysis of the FPN family in plants. A previous characterization of the family had a broader scope that did not allow an in-depth analysis of the family in plants (Schaaf et al., 2006). Here, we see a clear distinction between MAR1 and the FPN-like members of the family. The number of family members varies in distinct species. With the exception of *Kalanchoe fedtschenkoi* which lacks a putative *FPN* ortholog, plants have at least one copy of *MAR1* and one copy of *FPN*. 37 of 56 analyzed plants have one *MAR1* ortholog, while the rest have two to four. The presence of more than one copy of *MAR1* complicates its usage as a KOM for a species as either more alleles have to be mutated or the specific function of each gene must be elucidated first. For *FPN* orthologs the copy number was varying between zero and five with most species having either one or two. While the family is conserved in Brassicaceae with all species having two *FPN* copies, a different picture emerges when comparing sequences from other plant species. 30 of the 56 species only have one *FPN* ortholog, which is interesting as not only the localization, but also the function of AtFPN1 and AtFPN2 differ (Kobayashi and Nishizawa, 2012). Single copy FPNs either exhibit both functions of their *Arabidopsis* counterparts or there are other proteins that fulfill the missing role. We think that further characterization of this family in other plant species than *Arabidopsis* might improve the understanding of iron transport mechanisms in plants.

## Data Availability

The original contributions presented in the study are included in the article/Supplementary Material, further inquiries can be directed to the corresponding author.
