# Supplementary material for: Loss of MAR1 Function is a Marker for Co-Selection of CRISPR-Induced Mutations in Plants
Source: Front Genome Ed. 2021 Aug 9;3:723384. doi: 10.3389/fgeed.2021.723384 (PMC8525433; doi:10.3389/fgeed.2021.723384)
Supplement: Supplementary file 1 [file DataSheet1.PDF]

Supplementary Tables

Supplementary Table 1. Vectors used in this study.

Name	Description
V113	pGTR; Amplification of gRNA cassettes; Addgene: 63143
V117	pTC217; Geminiviral expression vector; Addgene: 70018
V118	pTC217 without homologous region and gRNAs, with multiple cloning site
V181	pICH47761 with napinA::turboGFP; Addgene: 48003
V182	pICH47742 with egg-cell specific Cas9 cassette; Addgene: 48001
V183	pICH47732 with phosphinotricin acetyltransferase expression cassette; Addgene: 48000
V184	pDI1; Shuttle vector modified from Addgene: 79461
V186	pDI2; Shuttle vector modified from Addgene: 79462
V187	pDI2E; Shuttle vector modified from Addgene: 79463
V188	pDI3; Shuttle vector modified from Addgene: 79465
V189	pDI4E; Shuttle vector modified from Addgene: 79466
H386	Tomato transformation vector based on V118 with gRNA 3 and 4
H387	Tomato transformation vector based on V118 with gRNA 1 and 2
H668	pDI1 with *AtMAR1* gRNA
H669	pDI2 with *AtMAR1* gRNA
H672	pDI3 with *At* target gRNA
H673	pDI4E with *At* target gRNA
H675	pICH47751 with the four *At* gRNA cassettes from H668, H669, H672 and H673; Addgene: 48002
H677	pAGM4723 with expression cassettes from V181, V182, V183 and H675; for Arabidopsis transformation; Addgene: 48015
H749	pDI1 with *AtMAR1* gRNA
H753	pDI2E with *At* target gRNA
H757	pICH47751 with the two *At* gRNA cassettes from H749 and H753; Addgene: 48002
H761	pAGM4723 with expression cassettes from V181, V182, V183 and H757; for Arabidopsis transformation; Addgene: 48015

Supplementary Table 2. Primers used in this study.

Name	Sequence	Description
P-274	TAGGTCTCCAAACGAAGACAAAAAC	pGTR general reverse
P-294	CGGGTCTCAGGCAGAAGACTAATTG	pGTR general forward
P-307	CCCACATCGCTTAGATAAGAAAACG	Sequencing H386 and H387
P-395	TAAGCCGGTCTCGGTTTCACGTGCTTCTAGAACCCGG GATGGAGCGAGACGAACGTCTCTCGCTATGACTAGT TCGACGTCAGATTTAAATGAGTGTACTTCAAGTC	Multiple Cloning Site
P-860	TAGGTCTCCGAAGGCGGACCAGTTTTAGAGCTAGAA	gRNA1 *SlMAR1* forward
P-861	ATGGTCTCACTTCCTTACTCTTGCACCAGCCGGGAA	gRNA1 *SlMAR1* reverse
P-862	TAGGTCTCCTTTACCACGCAAGTTTTAGAGCTAGAA	gRNA2 *SlMAR1* forward
P-863	ATGGTCTCATAAAGCGAGGAGTGCACCAGCCGGGAA	gRNA2 *SlMAR1* reverse
P-864	TAGGTCTCCTGGCAGCAGGTGGTTTTAGAGCTAGAA	gRNA3 *SlMAR1* forward
P-865	ATGGTCTCAGCCATTGCGTTGTGCACCAGCCGGGAA	gRNA3 *SlMAR1* reverse
P-866	TAGGTCTCCCTGGGTATATGGGTTTTAGAGCTAGAA	gRNA4 *SlMAR1* forward
P-867	ATGGTCTCACCAGCCAAGCAATGCACCAGCCGGGAA	gRNA4 *SlMAR1* reverse
P-1248	ATATTCACTGACCACACATC	Genotyping *SlMAR1*
P-1249	GAATCAACATCACTACTCAC	Genotyping *SlMAR1*
P-1250	CTGTTAGAGTGAAGACTACTACC	Genotyping *SlMAR1*
P-1251	GGAACCTCCTGTACAAAAAC	Genotyping *SlMAR1*
P-1436	ATTGGATAATTGCTGGAGGCCCTG	gRNA *AtMAR1* forward (H677)
P-1437	AAACCAGGGCCTCCAGCAATTATC	gRNA *AtMAR1* reverse (H677)
P-1661	ATTGAGCCTATGAGACTAGGCCTG	gRNA *At* target forward (H677)
P-1662	AAACCAGGCCTAGTCTCATAGGCT	gRNA *At* target reverse (H677)
P-1663	ATTGGCCTAAGCATGCGTTTGGAG	gRNA *At* target forward (H677 and H761)
P-1664	AAACCTCCAAACGCATGCTTAGGC	gRNA *At* target reverse (H677 and H761)
P-1665	ATTGGCCACTCCTGCTCACCCTGA	gRNA *AtMAR1* forward (H677 and H761)

P-1666	AAACTCAGGGTGAGCAGGAGTGGC	gRNA *AtMAR1* reverse (H677 and H761)
P-1780	GTAAAACGACGGCCAGTGCTTAGGATTTCGATGGGATCG	Capillary sequencing *At* target
P-1781	CTAACCACTGCAAACACAGCC	Capillary sequencing *At* target
P-1784	GTAAAACGACGGCCAGTCAGCATTGCCTGTACGTTC	Capillary sequencing *AtMAR1*
P-1785	GCATTGCAATGGCGGACG	Capillary sequencing *AtMAR1*
M13-FAM	GTAAAACGACGGCCAGT	Fluorescence-labelled (FAM) for AFLP
M13-JOE	GTAAAACGACGGCCAGT	Fluorescence-labelled (JOE) for AFLP

Supplementary Table 3. Different types of mutations induced by the CRISPR/Cas9-system in *MAR1* and the target gene.

Type of mutation	*MAR1*		Target gene	
	**Number of detected alleles**	**Percentage of all *MAR1* mutations**	**Number of detected alleles**	**Percentage of all target gene mutations**
Insertion > 2 bp	2	2.3	0	0
Insertion 1 bp	57	65.5	41	64.1
Deletion 1 bp	11	12.6	20	31.3
Deletion 2-10 bp	11	12.6	3	4.7
Deletion > 10 bp	6	6.9	0	0
**Total**	**87**	**100**	**64**	**100**

Supplementary Table 4. Putative orthologs of *AtMAR1* with a sequence identity higher than 50% of 56 plant species. Plant species used for phylogenetic analysis are highlighted in grey.

Family	Organism	*MAR1*-orth.	Locus tag
**Brassicaceae**	*Arabidopsis thaliana*	1	AT5G26820.1
	Arabidopsis halleri	1	Araha.25491s000
	Arabidopsis lyrata	1	AL6G38700.t1
	Brassica oleracea capitata	1	Bol012754
	Boechera stricta	1	Bostr.2128s0093.1
	Brassica rapa	1	Brara.B03574.1
	Capsella grandiflora	1	Cagra.0418s0004.1
	Capsella rubella	1	Carubv10000515m
	Eutrema salsugineum	1	Thhalv10003867m
**Embryophyte**	*Marchantia polymorpha*	1	Mapoly0001s0146.1
	Physcomitrella patens	2	Pp3c25_630V3; Pp3c5_21200V3
	Sphagnum phallax	1	Sphfalx0092s0074
**Tracheophyte**	*Selaginella moellendorffii*	1	62145 (gw1.11.772.1)
**Angiosperm**	*Ananas comosus*	1	Aco007359.1
	Amborella trichopoda	1	evm_27.model.AmTr_v1.0_scaffold00003.40
	Musa acuminata	2	GSMUA_Achr8T06360_001; GSMUA_Achr3T08330_001
	Spirodela polyrhiza	1	Spipo3G0069600
	Zostera marina	1	Zosma2g01370.1
**Grass**	*Brachypodium distachyon*	2	Bradi4g04670.2; Bradi1g67620.2
	Brachypodium stacei	2	Brast10G052700.1; Brast02G120800.1
	Oryza sativa	2	LOC_Os12g37530.1; LOC_Os05g04120.1
	Oropetium thomaeum	2	Oropetium_20150105_21861A; Oropetium_20150105_15432A
**Panicoideae**	*Panicum hallii*	2	Pahal.I04577.1; Pahal.C01213.1
	Panicum virgatum	4	Pavir.Ca02569.1; Pavir.Ca00756.1; Pavir.Cb00583.1; Pavir.J15949.1
	Setaria italica	2	Seita.3G335000.1; Seita.3G051000.1

	Setaria viridis	2	Sevir.3G349400.1; Sevir.3G051500.1
	Sorghum bicolor	2	Sobic.008G129900.1; Sobic.009G031000.1
	Zea mays	2	Zm00008a002625_T01; Zm00008a031796_T01
**Eudicot**	*Aquilegia coerulea*	1	Aqcoe5G312000.1
**Pentapetalae**	*Amaranthus hypochondriacus*	1	AHYPO_014711-RA
	Kalanchoe fedtschenkoi	2	Kaladp1071s0022.1; Kaladp0034s0193.1
	Kalanchoe laxiflora	4	Kalax.0461s0032.1; Kalax.0334s0009.1; Kalax.0084s0037.1; Kalax.0276s0018.1
**Asterid**	*Daucus carota*	1	DCAR_005423
	Mimulus guttatus	1	Migut.B00957.1
	Solanum lycopersicum	1	Solyc01g100610.2.1
	Solanum tuberosum	1	PGSC0003DMT400007140
**Rosid**	*Eucalyptus grandis*	1	Eucgr.L00261.1
	Vitis vinifera	1	GSVIVT01031249001
**Malpighiales**	*Linum usitatissimum*	4	Lus10029841; Lus10020698; Lus10031421; Lus10010912
	Manihot esculenta	1	Manes.08G110700.1
	Populus trichocarpa	1	Potri.005G012900.3
	Ricinus communis	1	30128.m008918
	Salix purpurea	2	SapurV1A.1038s0110.1; SapurV1A.2359s0010.1
**Citrus**	*Citrus sinensis*	3	orange1.1g007569m; orange1.1g010361m; orange1.1g015174m
	Citrus clementina	1	Ciclev10000649m
**Brassicales-Malvales**	*Carica papaya*	1	evm.model.supercontig_48.168
	Gossypium raimondii	2	Gorai.004G236200.1; Gorai.013G129700.1
	Theobroma cacao	1	Thecc1EG026774t1
**Fabidae**	*Cucumis sativus*	1	Cucsa.090580.1
	Fragaria vesca	1	mrna31598.1-v1.0-hybrid
	Glycine max	1	Glyma.10G146600.1
	Malus domestica	2	MDP0000294274; MDP0000150969
	Medicago truncatula	1	Medtr1g084140.1
	Phaseolus vulgaris	1	Phvul.007G201800.1
	Prunus persica	1	Prupe.6G312300.1
	Trifolium pratense	1	Tp57577_TGAC_v2_mRNA2710

Supplementary Figures

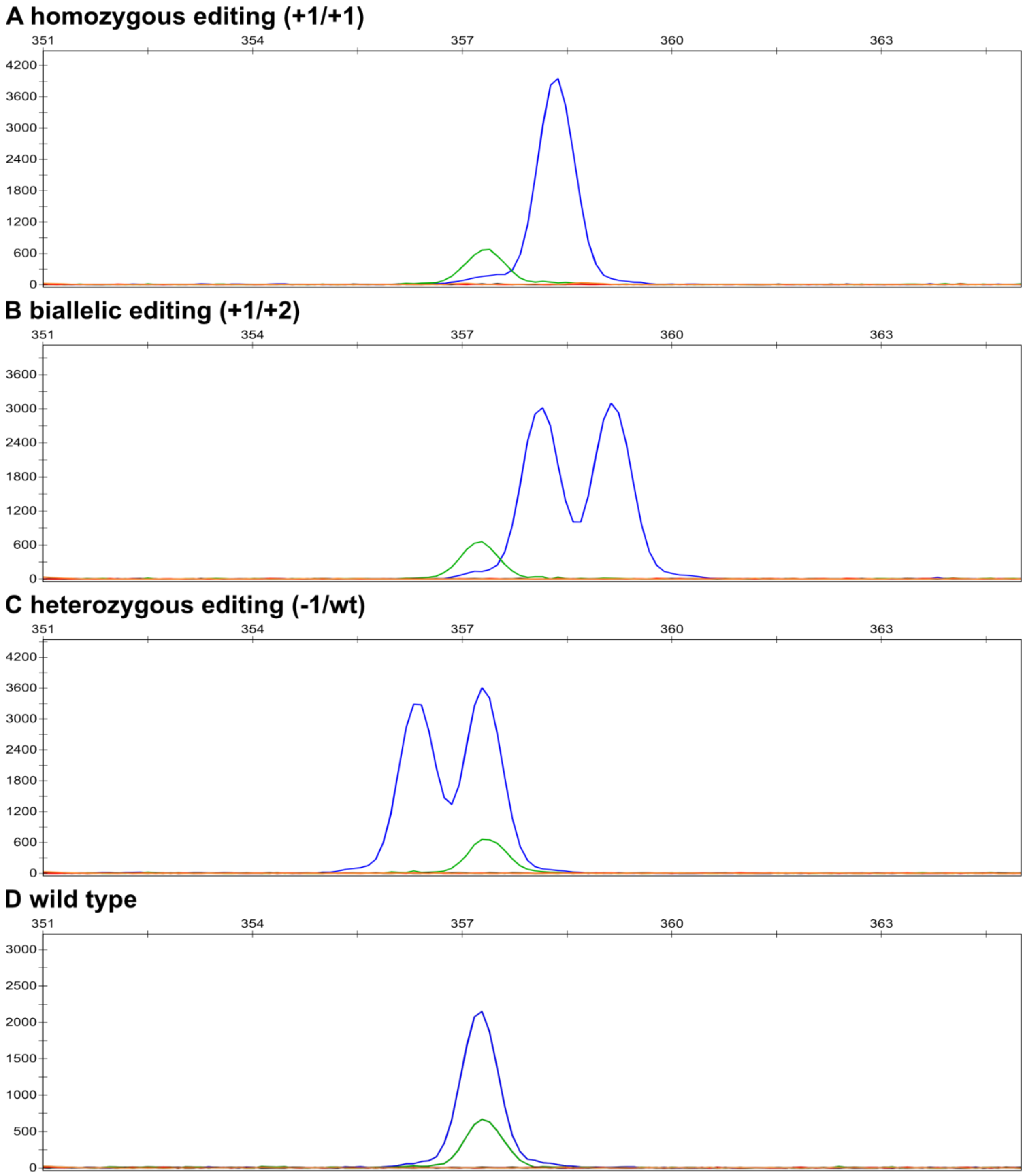

Supplementary Figure 1. Capillary sequencing chromatograms generated with GeneMapperID v3.2. The x-axis indicates the size of the amplicons, the y-axis the strength of detected fluorescence. Green peaks represent JOE-labelled amplicons of wild type plants, blue peaks are 6-FAM-labelled amplicons from H761-plants. Displayed are examples for a (A) homozygous, (B) biallelic, (C) heterozygous and (D) wild type mutation pattern. The numbers in the brackets indicate the number of inserted or deleted nucleotides.

```
AcoMar   1 LYASCFAGNVVEQLWNFAWPAAVALLHP-SLLPVAVVGFFTKLAIFIGGPLVGRLMDHFP
VviMar   1 LYASSFAGGLVEQLWNFAWPAAIALLHP-SLQPVAVIGFFTKLAVVVGGPLVGQLMDYFP
BraMar   1 LYASCLVGNLVEQLWNFAWPSAIAMLHP-SLLPVAVMGFVTKLAIIICGPVVGKFMDHSP
EsaMar   1 LYASCLVGNLVEQLWNFAWPSAIAMLHP-SLLPVAVMGFVTKLAIIIGGPVVGKFMDHSP
CgrMar   1 LYASCLVGNLVEQLWNFAWPSAIAMLHP-SLLPVAVMGFVTKLMIIAGGPVVGKFMDYSP
BstMar   1 LYASCLVGNLVEQLWNFAWPSAIAMLHP-SLLPVAVMGFVTKLVIIAGGPVVGKFMDYSP
AthMar   1 LYASCLVGNLVEQLWNFAWPSAIAMLYP-SLLPVAVMGFVTKLAIIAGGPVVGKFMDYSP
AlyMar   1 LYASCLVGNLVEQLWNFAWPSAIAMLHP-SLLPVAVMGFVTKLAIIAGGPVVGKFMDYSP
CsaMar   1 LYASCIAGNLVEQLWNFAWPSAIALLHP-SLLPVAVMGFFTKLALIVGGPLVGKFMDNFP
FveMar   1 LYASCLAGNLVEQFWNFAWPSAIALIYP-SLLPVALMGFVSKLAIIAGGPIVGTLMDHLP
PpeMar   1 LYASCLAGNLVEQFWNFAWPSAIALIYP-SLLPVAFMGFFSKLAIIAGCPLVGTLMDHFP
MesMar   1 FYASCLAGNLVEQLWNFAWPSAIALIHP-SLLPVAVMGFFTKLAIIVGGPLVGKLMDHSP
TcaMar   1 FYASCLAGNLVEQLWNFAWPSAIALLHP-SLLPVAVMGFFTKLVIIVGGPLVGKLMDHSP
MguMar   1 LYASCLAGNLVEQLWNFAWPAAISLIHP-SLLPVAVLGFFAKVAVIVGGPLVGKLMDYFP
SlyMar   1 LYASCLAGYMVEQLWNFASPAAIALIHP-SLLPVALMGFLAKLAVIGGGPLVGKLMDHFP
StuMar   1 LYASCLAGYMVEQLWNFASPAAIALIHP-SLLPVALMGFLAKLTVIVGGPLVGKLMDHFP
Bra3     1 LYVGYFLARWGARTWEFSVALYMIYLWPNSLLLAAIYGAIESGSTAIFGPIVGRWIEGMD
Esa3     1 LYVGYFLARWSARTWEFSVALYMIYLWPNSLLLAAIYGAIESGSTAIFGPIVGQWIEGMD
Ath3     1 LYVGYFLARWSARTWEFSVALYMIHLWPNSLLLAAIYGAIESGSTAIFGPIVGQWVEGMD
Aly3     1 LYVGYFLARWSARTWEFSVALYMIHLWPNSLLLAAIYGAIESGSTAIFGPIVGQWVEGMD
Bst3     1 LYVGYFLARWSARTWEFSVALYMIHLWPNSLLLAAIYGAIESGSTAIFGPVVGQWVEGMD
Cgr3     1 LYVGYFLARWSARTWEFSVALYMIHLWPNSLLLAAIYGAIESGSTAIFGPIVGKWVEGMD
Esa2     1 LYLGYFLARWGARTWEFSVALYMIYLWPNSLFLTAMYGAVESGSTAIFGPIVGQMIDGMS
Bra2     1 LYLGYFLARWGARTWEFSVALYMIYLWPNSLFLTAMYGAVESGSTAIFGPIVGQMIDGMS
Ath2     1 LYLGYFLARWGARTWEFSVALYMIYLWPNSLFLTAMYGVVESGSATLFGPIVGQMIDGMS
Aly2     1 LYLGYFLARWGARTWEFSVALYMIYLWPNSLFLTAMYGVVESGSAAIFGPIVGQMIDGMS
Bst2     1 LYLGYFLARWGARTWEFSVALYMIYLWPNSLFLTAMYGVVESGSTAIFGPIVGQMIDGMS
Cgr2     1 LYLGYFLARWGARTWEFSVALYMIYLWPNSLFLTAMYGAVESGSTAIFGPIVGQMIDGMS
Aco2     1 LYVGHFLSRWGARMWEFSVGLYMINIWPDSLLVTAIYGAAEAASTMLFGPIIGRLVDRLS
Mgu2     1 LYVGHFLARWGARMWEFSVGLYMINVWPDSLLLAAAYGVVESSSTALFGPLIGHWVDNLS
Sly2     1 LYVGHFLSRWSARMWEFSVGLYMINVWPDSLLLTAAYGVVESASTALFGPLVGQWVDRLT
Stu2     1 LYVGHFLSRWSARMWEFSVGLYMINVWPDSLLLTAAYGVVESGSTALFGPLIGQWVDRLT
Csa2     1 -------------MWEFSVGLYMISVWPNSLLFAAIYGVVESASTAFFGPIVGDLVDKLA
Fve2     1 LYIGHFLARWGARMWEFSVGLYMINLWPDSLLFAATYGVVESASTALFGPLVGQWVDKLS
Ppe2     1 LYVGHFLARWGTRMWEFSVGLYMISIWPDSLLFAAIYGAVESASTALFGPLIGHWVDRFP
Mes2     1 LYIGQFLARWDARMWEFCVGLYMITLWPNSLILPAIYGATESASIALFGPIVGQCAEKLT
Mes3     1 LYIAHFFARWDARMWEFSVGLYMITLWPDSLMLAAIYGAIESASTAFFGPIVGQWVERST
Mes4     1 LYIAHFFARWDARMWEFSVGLYMITLWPDSLMLAAIYGAIESASTAFFGPIVGQWVERST
Tca2     1 LYAAHFLARWGARMWEFSVGLYMISVWPDSLLLAAIYGAVESASTALFGSVIGRWVDRLT
Vvi2     1 LYVGHFLARWGARMWEFSVGLYMINIWPNSLLLAAVYGVVESASTAFLGPSIGQWLDRLT
Vvi3     1 LYVGHFLARWGARMWEFSVGLYMINIWPNSLLLAAVYGVVESASTAFLGPSIGQWLDRLT
```

```
AcoMar   60 RVPAYNCLSSVQAAAQLLSVAMIIHAHTVPSTAA--AMLLQPWFLVLVFAGAIERLSGLA
VviMar   60 RVPAYNCLNLVQATAHLLSVAMIIRAHTVPSTLA-SSVLLRPWFFVLVLAGSVERLSGLA
BraMar   60 RVPTYISLNVVQAAAQVLSAGMIIHAYTVPSTLG-SSILLQPWFFALIFAGAIDTLCGIA
EsaMar   60 RVPTYISLNVVQAAAQVLSAGMIIHAYTVPSTLG-SSILLQPWFFALIFAGAIDTLCGIA
CgrMar   60 RVPTYISLNVVQAAAQVLSAGMIIHAYTVPFPSA-SSILLQPWFFALVFAGAIDSLCGIA
BstMar   60 RVPTYISLNVVQAAAQVLSAGMIIHAYTVPSTSA-SSILLQPWFFALVFAGAIDSLCGIA
AthMar   60 RVPTYISLNVIQAAAQVLSAGMIIHAYTVPSTSA-SSILLQPWFFALLFAGAIDSLCGIA
AlyMar   60 RVPTYISLNVIQAAAQVVSAGMIIHAYTVPSTLA-SSILLQPWFFALLFAGAIDSLCGIA
CsaMar   60 RVPAYTCLNCVQAAAQLLSASMVIYAHTVPHTAASSSILLQPWFVTLIFAGAIERLSGIA
FveMar   60 RVPAYNCLNIIQAAAQLLSAAMIIRAHSVPP--SASSLLIRPWFIVLVLSGAVERLSGVA
PpeMar   60 RVPAYNCLNIIQATAQLLSAAMIIHAHSVPP--SVSSLLLRPWFIVLVLAGAVERLSGVA
MesMar   60 RIPSFIGLNVIQVAAQLLSATMIIRAHTVSPTSA-SSILLRPWFLVLVSVGAIERLCGVA
TcaMar   60 RVPSYIFLNVVQAAAQLLSASMIIHAHMVSPASA-SSVLRHPWFAVLVLAGAIERLSGVA
MguMar   60 RVPAYNCLTMVQGAAQLLSVGMIIHAHTIHPTPV-SSVLVQPWFVVLVIALAVERLCGLA
SlyMar   60 RVPAYNCLYIVQTAAQLMSVGMIIHGHTLHPTSA-SSLLFRPWFIVLVLVGAVERLSGLA
StuMar   60 RVPAYNCLSIVQTAAQLMSVGMIIHGHTLHPTSA-SSLLFRPWFIVLVLVGAVERLSGLA
Bra3     61 YVKVLRLWLLCQNLSYIIAGGAVIKLLLDYDLKPRNIPVFATLIALTNVAGAIGVLSTLG
Esa3     61 YVKVLRLWLLCQNLSYTIAGGAVIKLLLSPNLKSNNSSVFAILVALTNVSGAIGMLSTLG
Ath3     61 YVKVLRLWLLFQNLSYTIAGGAVIKLLLVSDLKSRNLPVFAILIVLTNLAGAIGVLSTLA
Aly3     61 YVKVLRLWLLFQNLSYTIAGGAVIKLLLVSDLKSRNLAVFAILVVLTNVAGAIGVLSTLA
Bst3     61 YVKVLRLWLLFQNLSYTIAGGAVIKLLLVSDLKSQNFPVFAILVVLTNIAGAIGVLSTLA
Cgr3     61 YVKVLRLWLLFQNLSYTIAGGAVVKLLLVSDLKSQNFLVFAILVVLTNVAGAIGVLSTLA
Esa2     61 YVKVLRLWLVTQNLSFIVAGVAIVALLLAPDLKSQNFPLFATLVVLTNLSGAVGVLSTLA
Bra2     61 YVKVLRLWLVTQNLSFIVAGGSVIALLLVPDLKSQNFPVFAALVVLTNLSGAIGVLSTLA
Ath2     61 YVKVLRLWLVTQNLSFIVAGGAVVALLVVPDLKSQNFPVFATLVVLTNLSGAIGVLSTLA
Aly2     61 YVKVLRLWLVTQNLSFIVAGGAVVALLLVPDLRSHNFPVFAILVVLTNVSGAIGVLSTLA
Bst2     61 YVKVLRLWLVTQNLSFIVAGGAIVALLLVPDLKSQNFPVFATLLVLTNLSGAIGVLSTFA
Cgr2     61 YVKVLRLWLVTQNLSFIVAGGAVVALLVVPDLKSQNFPVFATLVVLTNLSGAIGVLSTLA
Aco2     61 YIQVLRLWLFIQNISFIVAGGTVTALLLYPGLRISHFPLFISLIIITNISGAIGVLATLA
Mgu2     61 YIQVLRIWLLSQNLSFMVAGGAVVAILAYPNSISANYTSFISLVLLINISGAVGVLSTLG
Sly2     61 YVKVLQLWLLSQNISFIVAGGAVIALLVRADLILVNLTAFISLVSLIYISGAVGVLSSLA
Stu2     61 YVKVLQLWLLSQNISFIVAGGAVIALLVRADLILVNLTAFISLVSLIYISGAVGVLSSLA
Csa2     48 YVKVLKIWLATQNLSYIVAGVTVVALLFYSDLKSSYFTGFILLVILTNIAGAVGALSSLA
Fve2     61 YVKILRLWLVTQNLSFMIAGGTVMGLLIYPDLKLTNFSAFVSLIILTNISGALGVLSTLA
Ppe2     61 YVKVLWLWLVTQNLSFMIAGGTVMALLVYSDLKLTSFNAFVLLVILTNISGAVGVLSTLA
Mes2     61 YVKVLRIWLVTQNLSFVIAGLSVIALLVFSTLKSTNFTAFIFLVILTNISGAIGVLSTLA
Mes3     61 YVKVLRIWLVTQNLSFMVAGCTVIALIVFSTLKTTNFTAFILLVILTNISGAVGVLSTLA
Mes4     61 YVKVLRIWLVTQNLSFMVAGCTVIALIVFSTLKTTNFTAFILLVILTNISGAVGVLSTLA
Tca2     61 YVKVLKLWLVTQNLSLIIAGCAVMALLVFSSLKVTNLVAFISLVILTNISGAVGVLSTLA
Vvi2     61 YVKVLKLWLWAQNLSFVVAGVAVVGLLVYSNLKYTNLAAFIALVILTNISGAIGVLSTLA
Vvi3     61 YVKVLQLWLWTQNLSFVVAGAAVVGLLFFSSLKHTNLAAFITIVALTNISGAVGVLSTLA
```

```
AcoMar   118 LGVAMERDWVVLLAGTNR-PIA-LAEANAIISRIDLICEIVGASLFGILLSKYDPVSCLK
VviMar   119 LGVTVERDWIVLLAGPNR-PIA-LAEANAVLNRIDLVCEIAGASLFGILLSKYDIMTYLK
BraMar   119 SGVAIERDWVVLLAGINR-PIA-LAQANAVLNRIDLLCEIAGTMLFGILLSKYDPVTCLK
EsaMar   119 SGVAIERDWVVLLAGINR-PIA-LAQANAVLNRIDLLCEIAGTTLFGILLSKYDPVTCLK
CgrMar   119 SGVAIERDWVVLLAGINR-PIA-LAQANAVLHRIDLLCEIAGTMLFGILLSKYDPVTCLK
BstMar   119 SGVAIERDWVVLLAGINR-PIA-LAQANAVLHRIDLLCEIAGTMLFGILLSKYDPVTCLK
AthMar   119 SGVAIERDWVVLLAGINR-PIA-LAQANAVLHRIDLLCEIAGTMLFGILLSKYDPVTCLK
AlyMar   119 SGVAIERDWVVLLAGINR-PIA-LAQANAVLHRMDLLCEIAGTMLFGILLSKYDPVTCLK
CsaMar   120 LGVAMERDWVVLLAGINR-PIA-LAEANAVLSRIDLLCEIVGASLFGIILSKYDPVTCLK
FveMar   118 LGVAAERDWVVQLAGVNR-PIA-LAQANAILSRIDLLSEIAGASLFGILISKYDPVTCLK
PpeMar   118 LGVAMERDWVVLLAGVNR-PIA-LAQANAIINRIDLLCEIAGASLFGILLSKYDPVTCLK
MesMar   119 LGVAMERDWVVLLAGINR-PIA-LAQANAVLNRIDLLCEIAGASLFGILLSKYDPVTCLK
TcaMar   119 LGVAMERDWVVLLAGINR-PIA-LAQANAVLNRINLLCEIAGTLLFGILLSKYDPVTCLK
MguMar   119 LGVAMERDWVVLLAGTSR-PIA-LAQANAILSRIDLLCEIAGASLFGIFLSQHEPVTCLK
SlyMar   119 LGVAVERDWVVLLAGTNR-PVA-LAQANAVLSRINLLCEIVGAALFGILLAKYELVLCLK
StuMar   119 LGVAVERDWIVLLAGTNR-PVA-LAQANAVLSRINLLCEIVGAALFGILLSKYELVVCLK
Bra3     121 GTILIERDWAVVMSEGHP-PAV-LTRMNSVIRGIDLSSKLLSPVITGLIIS---FVSLKA
Esa3     121 GTILIERDWAVVMSEGHP-PGV-LTRMNSVIRGIDLSSKLLSPVITGLIIS---FVSLKA
Ath3     121 GTILIERDWAVVMSEGHP-PAV-LTKMNSVIRGIDLSSKLLSPVITGLIIS---FVSLKA
Aly3     121 GTILIERDWAVVMSEGHP-PAV-LTRMNSVIRGIDLSSKLLSPVITGLIIS---FVSLKA
Bst3     121 GTILIERDWAVVMSEGHP-LAV-LTRMNSVIRGIDLSSKLLSPVITGLIIS---FVSLKA
Cgr3     121 GTILIERDWAVVMSEGHP-PAV-LTRMNSVIRGIDLSSKLLSPVITGLIIS---FVSLKA
Esa2     121 GTILIERDWVVVMSEGHS-PAV-LTRMNSVIRSIDLSSKLLSPVITGLIIS---FVSLRA
Bra2     121 GTILIERDWVVVMSEGHS-PDV-LTRMNSVIRGIDLSSKLLSPVITGFIIS---FVSLEA
Ath2     121 GTVLIERDWVVVMSEGHS-PAV-LTRMNSVIRGIDLSSKLLSPVITGLIIS---FVSLRA
Aly2     121 GTILIERDWVVVMSEGHS-PTV-LTRMNSVIRGIDLSSKLLSPVITGLIIS---FVSLKA
Bst2     121 GTILIERDWVVVMSEGHS-PAV-LTRMNSVIRGIDLSSKLLSPAISGLLIS---FVSLRA
Cgr2     121 GTILIERDWVVVMSEGHS-PAV-LTRMNSVIRGIDLSSKLLSPVVTGLIIS---FVSLRA
Aco2     121 GTILVEREWVVVISNGQP-PEA-LTQMNSVIRRIDLVCKLFAPVVSGFIIS---FISLTD
Mgu2     121 GTILIEREWVVVISEGQP-PEV-QTKMNSTIRRIDLTCKLFAPVVSGFIIS---FVSLTA
Sly2     121 GTILIEREWVVVISEGHP-PGL-LTKMNSIIRRIDLICKLFAPVVTGFIIS---FVSLTA
Stu2     121 GTILIEREWVVVISEGHP-PGL-LTKMNSIIRRIDLVCKLFAPVITGFIIS---FVSLTA
Csa2     108 GTILVEREWVVVISERHP-PEV-LTNINSTMRRIDLVCKLLSPVISGFIIS---FISLKA
Fve2     121 GTILVERDWVVVISEGHS-PEV-LTNMNSIIRRIDLFSKLCAPVVTGFIIS---FVSLKA
Ppe2     121 GTILVERDWVVVISECHSDPEVLLTKMNSVLRRIDLFCKLCAPVLTGFIIS---FVSLKA
Mes2     121 GTILIEREWVVIISEGHP-PHV-LTKMNSIIRRIDLTCKLLAPVVAGSIIS---FLSTKA
Mes3     121 GTILIEREWVVVISEGQP-PGV-LTNMNSVIRRIDLTCKLLAPVVSGFIIS---FISVKA
Mes4     121 GTILIEREWVVVISEGQP-PGV-LTNMNSVIRRIDLTCKLLAPVVSGFIIS---FISVKA
Tca2     121 GTILIEREWVVVISEGHP-PGL-LTEMNSVIRRIDLTCKLVAPVITGFIIS---FVSLKA
Vvi2     121 GTILIEREWVVVISEGHP-PGV-LTKLNSVIRRIDLTCKLFAPVVTGLLIS---FVSLKA
Vvi3     121 GSILIEREWVVVMSEGHP-PDV-LTRMNSVLRRIDLTCKLFAPVVTGFIIS---FVSLKA
```

```
AcoMar  176 LAAGLMIATVPVVILLMWLSNKLSSGVLDRSKSPQAGK----MFNDMPKAGNLVEMGLDS
VviMar  177 FTAGLMTWTVPVVLVLTWLTNKLSSGVLNRTKSH--------------DAEVTVKIGVGA
BraMar  177 FAATLMMGSLPTMTALIWLTNKFSSGVLDRPKCSQSSCA---SEGPRSETESIFDLGMEA
EsaMar  177 FAATLMMGSLPTMTALIWLTNKFSSGVLDRPKCSQSNCA---TEAPRSDNKSIVDIGMEA
CgrMar  177 FAATLMVGSLPTMTALIWLTNKFSSGVLDRPKCSQGSYA---FEGPRSATNSISDI-VET
BstMar  177 FAATLMMGSLPTMTALIWLTNKFSSGVLDRPKCSQSSCA---AEGPRSATDSIFDIGMET
AthMar  177 FAATLMVGSLPTMTALIWLTNKFSSGVLDRPKCSLNSCS---AEGSRTNTDSIFDIGMET
AlyMar  177 FAATLMVGSLPTMTALIWLTNKFSSGVLDRPKCSLSSCS---AEGSRTNTDSIFDIGMET
CsaMar  178 CAAGLMIWSLPVVVLLTWLTNQLSTGVLDRAKCLQTCC------GDPTEVTSPVNVGVEV
FveMar  176 FATCLMVWSLPFAVFLTCLTNKLSSGVLDRPKCSQTCCRTS-TEGPLLDTNNIVDKGVEV
PpeMar  176 FAGGLMVWSLPFAIVLTCLTNKLSSGVLDRPKCSQTCCKAS-TEGPLIDTNDILDKGVAA
MesMar  177 FATGLMIWSLPIMIGLALLTNKLSTGVLDHTRFSQTCCRE--SNGTAVDDNSLVDRGLET
TcaMar  177 FAAGLMMGSLPVMISLTWLTNKLSTGVLDRARCSQSCCRTS-DEGQLPDADNFVNTGLEA
MguMar  177 LAAGLMIWSLPVVVVLTWLTNMLSAGVLDRAKCSQCCCGSS-SADSIPDTSNIFATSVEV
SlyMar  177 IAAGLMMGTLPIVVSLTWLTNKLSSGVLDRAVETCLSC----SFPSSLKSENIVGVGLEA
StuMar  177 IAAGLMMGALPIVVSLTWLTNKLSSGVLDRAVETCLSC----SFPSSLKSENIVGVGLEA
Bra3    176 SAITFAFWAIITAWVEYWLFISVYSGVPAIAQSNERRILRS-ITRPVEETDAPVSVSIVP
Esa3    176 SAITFAAWATITAWVEYWLFISVYIGVPAIIRSNERRILRS-RTKPVEGKDAAVPVCIEE
Ath3    176 SAITFAAWATITAWVEYWLFISVYSGVPAITRSNERRILRS-RTKQVEGRDAPVSVSIVP
Aly3    176 SAITFAAWATITAWVEYWLFISVYSGVPAIARSNERRILRS-RTKQVEGTDAPVSVSNAP
Bst3    176 SAITFAAWATLTAWLEYWLFISVFSGVPAIARSNEGRILRS-RTKPEEGTDAPVAISTVP
Cgr3    176 SAITFAAWATITAWLEYWLFISVYSGVPAIARSNERRILRS-RTDPVEETDAPVAISTVL
Esa2    176 SAITFAAWATITVWIEYWLFISVYSGVPAIVQSDARRSSRLMSRTQAEETDIASRYLHVP
Bra2    176 SAVTFAAWATITVWIEYWLFISVYNGVPAVLQSDERRSLRL-----SSQTDIASQYYVPL
Ath2    176 SAITFAAWATITVWIEYWLFISVYNGVPAIVQSDERRSLRS-SQSQAEETDSASSFYVPL
Aly2    176 SAITFAAWATITVWVEYWLFISVYNGVPAIVQSDERRRLRL-SQSQGEETESASSFYVPL
Bst2    176 SAIAFAAWATITVWIEYWLFISVYNGVPAIVQSDDRRSLRL-SQSQAEETDIASSYYVPL
Cgr2    176 SAITFAGWATITVWIEYWLFISVYNGVPAIVQSNERRSLRL-SQSLAEETDFASSYDVPL
Aco2    176 SAVTFTLWNIVSVSVIYWLLSSVYNGIPALIENSQKRNARF-PPNDSIESPPSLQETESL
Mgu2    176 SAVTLALWNVLSVFLQYWLLMSVYKGIPILRQFSQRRVSKS-SVMEVDESTSTHQEQDES
Sly2    176 SAMTLALWNVISVCLEYWLLTSAYNGIPALSESSLRRVSRS-LPEH-SDLSPSISHEQKS
Stu2    176 SAMTLALWNVISVFLEYWLLTSVYNGIPALSESSLRRASRS-SPEH-SDLSPTISHEQKS
Csa2    163 SAMTLAVWNIISVWLEYWLFTSVYDGIPALEESSQRRVSRL-ALGDVGESSSVSQQIERL
Fve2    176 SALTLALWNTISVWLEYWLFISVFNGIPALGESSQRKITRP-SQSDLEGIGASTSQERSS
Ppe2    178 SALTLALWNTISIWLEYWLFISVYNGIPALGESSQRKISRP-SRTDVEESTSTSDQERIS
Mes2    176 SAMALAFWNMSAVWMEYWFFTSVYKGIPALGEGSRRRNSRL-SPSNMEEGVPTSTKPENL
Mes3    176 SAMTLALWNTIAVWMEYWLFTSVYKGIPALGESSQRKVLRF-SHSDHEETASLSSQQASL
Mes4    176 SAMTLALWNTIAVWMEYWLFTSVYKGIPALGESSQRKVLRF-SHSDHEETASLSSQQASL
Tca2    176 SAMTLALWTTITVWVEYWLFMSVYNGIPALGESSLRRISEV-SPSDVAEESTSASQEIPD
Vvi2    176 SAMTLAIWNTLSIWLEYWLLTSVYNGIPALSERSQKKISKI-SQGDPGESTSADQEIKSS
Vvi3    176 SAMTLAIWNTVSVWLQYWLLMSVYNGIPALRESNQKRTSKIAERSAGESTSACQGVNSSP
```

```
AcoMar  232 IKH------------------------------GWIEYKQQPVLPASLAYVLLYFNVVLT
VviMar  223 IKH------------------------------GWMEYLQQPVLPASLAYVLLYFN-VLM
BraMar  234 IKL------------------------------GWKEYIQQPVLPASLAYVLLCFNIVLT
EsaMar  234 IKL------------------------------GWKEYIQQPVLPASLAYVLLYFNIVLT
CgrMar  233 IKL------------------------------GWKEYIQQPVLPASLAYVLLYFNIILT
BstMar  234 IKL------------------------------GWKEYIQQPVLPASLAYVLLYFNIVLT
AthMar  234 IKL------------------------------GWKEYIQQPVLPASLAYVLLYFNIVLT
AlyMar  234 IKL------------------------------GWKEYIQQPVLPASLAYVLLYFNIVLT
CsaMar  232 IKN------------------------------GWKEYLQQPALPASLAYVLLYFNAVLA
FveMar  235 IKL------------------------------GWKEYLQQPVLPASLAYVLLYFNVVLT
PpeMar  235 IKL------------------------------GWKEYMQQPVLPASLAYVLLYFNVVLT
MesMar  235 IKL------------------------------GWKEYMHQPVLPASLAYVLLCFNVVLA
TcaMar  236 IKL------------------------------GWREYIQQPVLPASLAYVLLYLNVVLT
MguMar  236 IKN------------------------------GWFEYIQQPVLPASLAYVLLYFNVVLA
SlyMar  233 IKH------------------------------GWFEYIRQPVLPASIAYVLLYFNVVLA
StuMar  233 IKH------------------------------GWFEYIRQPVLPASIAYVLLYFNVVLA
Bra3    235 GTE------EGNPPRRTAMLKVFDRVSKSSFVGAWRVYIKQEVVLPGVSLALLFFT-VLS
Esa3    235 GSQ-------GNPPCRTGMLKILDRISESSFVGAWRIYINQEVVLPGVSLALLFFT-VLS
Ath3    235 GTE---EGYTGNPPSRTGILVILDRMSKSSFVGAWRIYFNQEVVLPGVSLALLFFT-VLS
Aly3    235 GTE---ESSTGNPPCRTGIRKILNRVSKSSFVSAWRIYFNQEVVLPGVSLPLLFFT-VLS
Bst3    235 GTE---EGSQGTPPRRIGILKIVDRISDSSFVGAWRIYFNQEVVLPGVSLALLFFT-VLS
Cgr3    235 RTE---EGSQGPPPCRTGILKIVDRISNSSFVGAWRIYFNQEVVLPGVSLALLFFT-VLS
Esa2    236 LLQ--GTTSEDNIPSRSGIMRILERISGSSFVAAWRNYLNQEIVLPGVALALLFFT-VLS
Bra2    231 LTD-------QNTRSRSGNMRILERISDSSFVNAWRNYLNQDIVLPGVALALLFFT-VLS
Ath2    235 LHE---EESYRNTQSRSRILRILERISESSFVSAWRNYLNQEIVLPGVSLALLFFT-VLS
Aly2    235 LHE---EESYRKSQSRSGIFRILERISESSFVSAWRNYLNQEIVLPGVALALLFFT-VLS
Bst2    235 LKGTTEDESYRNTQRGSGILRILERIFESSFVSAWRNYLNQEIVLPGVALALLFFT-VLS
Cgr2    235 LQGTTEDESYGNTQRRSGILRILDRISESSFVGAWRNYLNQEIVLPGVALALLFFT-VLS
Aco2    235 LPH--------KGSDSAVRHHLIERVSKIPYVQAWTVYLQQDVVLPGIALALLFFT-VLS
Mgu2    235 SSS--------------KGTTIIERFLNLPYISAWILYFKQDVVLPGLSLALLYFT-VLS
Sly2    234 SLH--SDGIGLEQSENLW-RKIVGSISRLPCLSAWKVYLQQDVVLPGLALALLFFT-VLS
Stu2    234 TFH--LDRIGLEQSENLW-RKIVGSISRLPCLSAWKVYLQQDVVLPGLALALLFFT-VLS
Csa2    222 IPN----DVDARSAERSWKVKMFNWFSKVPFVTAWKVYLEQDTVLPGVALALLFFT-VLS
Fve2    235 LLS--KDENDSDFAKESWITRVIKMVSRMPYVNAWSVYLQQDVVLPGVSLALLFFT-VLS
Ppe2    237 LLS--HDENDSQPAEESWIKRITEAVSRIPYVGAWKVYLQQDVVLPGVALALLFFT-VLS
Mes2    235 HSQ---NGENSALMERKWRRKLTVWVSKAPFVGAWKVYLQQDVLLPGVALALLYFT-VLS
Mes3    235 LSQ---NEETSALEDKSWRKKLSEWISQAPFLGAWSTYLQQDVAVPGVALALLYFT-VLS
Mes4    235 LSQ---NEETSALEDKSWRKKLSEWISQAPFLGAWSTYLQQDVAVPGVALALLYFT-VLS
Tca2    235 SLS--NNGENSAAAGKNSGSKFIEWVAKVPYIDAWRVYLQQDVVLPGVALALLYFT-VLS
Vvi2    235 PSF---DGGDSALAENSWKRKMIEWVWKALSISAWTVYLQQDVVLPGLALALLYFT-VLS
Vvi3    236 LAD------------NSWKRKMIKCVWKVLCISAWNVYLRQDVVLSGVALALLYFT-VLS
```

```
AcoMar   262 PGGLMTAFLTQRGLDPSIIGGFSGLCAFMGVAATFISATLVKRLGILKAGAAGLVFQASL
VviMar   252 PGGLMTAFLTQRGLNPSIVGGFSGLCAFMGVAATFISANLVRRLGMLKAGAAGLIFQAFL
BraMar   264 PGSLMTAFLTQRCVNPSVIGGFTGLCAVMGVAATFLSANLVKRFGILKAGAVGLFFQASL
EsaMar   264 PGSLMTAFLTQRCVNPSVIGGFSALCAVMGVAATFLSANLVKRFGILKAGAVGLFFQASL
CgrMar   263 PGSLMTAFLTQRSVNPSVIGGFSGLCAVMGVAATFLSANLVKRFGILKAGAVGLFFQASL
BstMar   264 PGSLMTAFLTQRCVNPSVIGGFSGLCAVMGVAATFLSANLVKRFGILKAGAVGLFFQASL
AthMar   264 PGSLMTAFLTQRCVNPSVIGGFSGLCAVMGVAATFLSANLVKRVGILKAGAVGLFFQASL
AlyMar   264 PGSLMTAFLTQRCVNPSVIGGFSGLCAVMGVAATFLSANLVKRVGILKAGAVGLFFQASL
CsaMar   262 PGSLMTAFLTQQGLSPSIIGGFSGLCAFMGVTATFVSANLVRQFGILKAGAVGLIFQAAL
FveMar   265 PGSLMTAYLTQSGLNPSIIGGFSGLCAFMGVAATFVSASLVRRLGILKAGAAGLIIQASL
PpeMar   265 PGSLMTAYLTQSGLNPSIIGGFSGLCAFMGVAATFVSSCLVRRLGILKAGAAGLIFQASL
MesMar   265 PGSLMTAFLTQRGVNPSIIGGFSGLCASMGVVATFLSATLVKRLGILKAGAAGLVFQASL
TcaMar   266 PGSLMTAFLTQRGLNPSVIGSFSGLCAFMGVGATFLSATLVRRFGILKAGAVGLIFQASL
MguMar   266 PGSLMTAFLTQHGLNPTLIGGFSGLCAFMGVAATFVSAKMVKRLGILKAGAAGLIFQASL
SlyMar   263 PGGLMTAFLTQQGLNPSIIGGFSGLCAFVGVAATFVSAKMVKHLGILKAGAAGLVFHASL
StuMar   263 PGGLMTAFLTQQGLNPSIIGGFSGLCAFVGVAATFVSAKMVKHLGILKAGAAGLVFHASL
Bra3     288 FGTLMTATLQWEGIPTYIIGIGRGISATVGLAATVVYPLMQSRISTLRTGLWSFWSQWSC
Esa3     287 FGTLMTATLQWEGIPTYIIGIGRGISATVGLAATVVYPLMQSRISTLRTGLWSFWSQWSC
Ath3     291 FGTLMTATLQWEGIPTYIIGIGRGISATVGLAATLVYPLMQSRLSTLRTGLWSFWSQWSC
Aly3     291 FGTLMTATLQWEGIPTYIIGIGRGISATVGLAATLVYPLMQSRLSTLRTGLWSFWSQWSC
Bst3     291 FGTLMTATLQWEGIPTYIIGIGKGISATVGLAATFVYPLMQSRLSTLRTGLWSFWSQWSC
Cgr3     291 FGTLMTATLQWEGIPTYIIGIGRGISAMVGLAATFLYPLMQSRLSTLRTGLWSFWSQWSC
Esa2     293 FGTLMTATLEWKGIPTYIIGIGRGISAGVGLAATVVYPLMQSRLSPLRTGLWSFWSQWTC
Bra2     283 FGTLMTATLEWKGIPTYIIGIGRGISAGVGLTATVVYPLLLSRLSPLRTGLWSFWSQWTC
Ath2     291 FGTLMTATLEWKGIPTYIIGIGRGISAGVGLAATVLYPLMQSRISPLRTGVWSFWSQWTC
Aly2     291 FGTLMTATLEWKGIPTYIIGIGRGISAGVGLAATVLYPLMQSRISPLRTGLWSFWSQWTC
Bst2     294 FGTLMTATLEWKGVPTYIIGIGRGISAGVGLAATVVYPLMQSRLSSLRTGLWSFWSQWTC
Cgr2     294 FGTLMTATLEWKGIPTYIIGLGRGISAGVGLAATVVYPLMQSRLSPLRTGLWSFWSQWTC
Aco2     286 FGTLMTATLQWEGIPAYVIGIMRGISATIGIAATLLYPILHSRISTLRTGLWSIWMQWTF
Mgu2     280 FGTLMTAALEWQGIPAYVIGIARGISATVGIAATFLYPVLQSRMSTLRTGLWSIWSQWTC
Sly2     290 FGTLMTATLEWEGIPAYVIGIARGVSATIGIAATFLYPILESHISTLRTGLWSIWSQWTF
Stu2     290 FGTLMTATLEWEGIPAYVIGIARGVSATIGIAATFLYPILESHISTLRTGLWSIWSQWTS
Csa2     277 FGTLMTATLEWEGIPAYIIGIARGVSATIGIAATLVYPIVQSRILTLRTGLWSIWSQWTC
Fve2     292 FGTLMTATLEWEGIPVYTIGIARGISAAIGITATIVYPILQSHILTLRTGLWSIWSQWTF
Ppe2     294 FGTLMTATLEWQGTPAYAIGIARGISAAIGIAATIVYPVLQSHILTLRTGLWSIWSQWTF
Mes2     291 FGILMTAALKWEGVPAFVIGIGRGISAIIGIGATVVYPILQSRILTLRTGLWSIWSQWSC
Mes3     291 FGTLMTATLEWEGIPALVLGIARGASALIGIGATLVYPILQSHIHTVRTGLWSIWSQWSC
Mes4     291 FGTLMTATLEWEGIPALVLGIARGASALIGIGATLVYPILQSHIHTVRTGLWSIWSQWSC
Tca2     292 FGTLMTAALEWEGIPAFVIGIARGISASIGIAATVVYPMLQSRILTLRTGLWSVWSQWNC
Vvi2     291 FGTLMTAALEWEGIPAYIIGIGRGISATIGIAATFVYPILQSRISILRTGLWSIWSQWAF
Vvi3     283 FGTLMTVALQWEGIPAYVIGIARGISAAIGIAATFVYPILQSHISTLRTGLWSIWSQWAF
```

```
AcoMar   322 LVLAVVVYWSGSLSRRTPLLFFLCLIVLSKLGHMSYDVVGSQILQTGIPASKANLIGTTE
VviMar   312 LTIAVAVYWSGSLSQQTPLLFFLCLIVLSRLGHMSYDVVGTQILQTGIPSSKANLIGTTE
BraMar   324 LGVAVAVYWSSSLSQKSPLFFFLSMIVLSRLGHMSYGVVGAQILQTGIPSSKANLIGATE
EsaMar   324 LGVAVAVYWSSSLSQKSPLFFFLSMIVLSRLGHMSYGVVGAQILQTGIPSSKANLIGATE
CgrMar   323 LAVAVTVYCSSSLSHKSPLFFFLSMIVLSRLGHMSYGLVGAQILQTGIPSSKANLIGATE
BstMar   324 LAVAVTVYCSSSLSQKSPLFFFLSMIVLSRLGHMSYGVVGAQILQTGIPSSKANLIGATE
AthMar   324 LAVAVAVYCSSSLSHKSPLFFFLSMIVLSRLGHMSYGVVGAQILQTGIPSSKANLIGATE
AlyMar   324 LAVAVAVYCSSSLSQKSPLFFFLSMIVLSRLGHMSYGVVGAQILQTGIPSSKANLIGATE
CsaMar   322 LTVAVAVYLSGSLSRQSPLLFFLVMIVLSRLGHMSYNVVGQQILQTGIPSSKTNLIATTE
FveMar   325 LTVAVAVYCSRSASQQSPLLFFLCLIVLSRLGHMSYDVVAAQILQTGIPPSKVNIIGTTE
PpeMar   325 LTAAVAVYCSRSSSQQSPLLFFLCLIVLSRLGHMSYDVVAVQILQTGIPSSKANIIGTTE
MesMar   325 LILAVAVYWSGSLSRQSPLLFFLGLIVLSRLGHMSYDVVGAQILQSGIPSSKANLIGATE
TcaMar   326 LTIAVAVYQSGSLSQKGPLLFFLCLIVLSRFGHMSYDIVGAQILQTGIPSSKANLIGTTE
MguMar   326 LTLAVAVYWSGSLSQKVPVLFFLCLIVLSRLGHMSYDVVGAQILQTGIPASKVNLIGTTE
SlyMar   323 LTTAVAVYWSGCLSQQTPVFFFLALVVLSRLGHMSYDVIGQQILQTGIPASKANLIGTTE
StuMar   323 LTTAVAVYWSGCLSQQTPIFFFLALVVLSRLGHMSYDVIGQQILQTGIPASKANLIGTTE
Bra3     348 LLVCVGSIWVK--RDNVASYMLMGGVAASRLGLWMFDLAVIQQMQDQVSESDRCVVGGVQ
Esa3     347 LLVCVGSIWVK--KDNIGSYMLMAGVAASRLGLWMFDLAVIQQMQDLVSESDRCVVGGVQ
Ath3     351 LLVCVGSIWVK--KDKIASYMLMAGVAASRLGLWMFDLAVIQQMQDLVSESDRCVVGGVQ
Aly3     351 LLVCVGSIWVK--KDKIASYMLMAGVAASRLGLWMFDLSVIQQMQDLVSESDRCVVGGVQ
Bst3     351 LLVCVGSIWVK--RDKIASYMLMAGVAASRLGLWMFDLAVIQQMQDLVSESDRCVVGGVQ
Cgr3     351 LLVCVGSIWVE--KDKIASYMLMAGVAASRLGLWMFDLAVIQQMQDLVSESDRCVVGGVQ
Esa2     353 LLVCVGSIWVE--TEKIASYMLMAGVAASRLGLWMFDLAVIQQMQDLVPESDRCVVGGVQ
Bra2     343 LLVCVGSIWVQ--KEKVASYMLMAGVAASRLGLWMFDLAVIQQMQDLVPESDRCVVGGVQ
Ath2     351 LLVCVGSIWVE--KEKIASYMLMAGVAASRLGLWMFDLAVIQQMQDLVPESDRCVVGGVQ
Aly2     351 LLVCVGSIWVE--KEKIASYMLMAGVAASRLGLWMFDLAVIQQMQDLVPESDRCVVGGVQ
Bst2     354 LLVCAGSIWVE--KEKLASYMLMSGVAASRLGLWMFDLAVIQQMQDLVPESDRCVVGGVQ
Cgr2     354 LLVCVGSIWVE--KEILASYMLMSGVAASRLGLWMFDLAVIQQMQDLVPEPDRCVVGGVQ
Aco2     346 LLVCVASVWVR--NSYLSAWLLMGGVAASRLGLWMFDLSVMQQMQDHVRESDRCVVGGVQ
Mgu2     340 LLLCVASVLVK--GKMVSAYMLMGGVALSRLGLWMFDLSVIQQMQDHVAESDRCVVGGAQ
Sly2     350 LLICVASIWVH--KKFLSAFMLMAGVAASRLGLWMFDLAVIQQMQDHVPESDRCVVGGVQ
Stu2     350 LLICVASIWVH--KKFLSAFMLMAGVAASRLGLWMFDLAVIQQMQDHVPESDRCVVGGVQ
Csa2     337 LLVCVVSIWIQ--NSLLSAYMLMMGVAVSRLGLWMFDLAVIQQMQDQVPESDRCVVGGAQ
Fve2     352 LLLCVASIWIH--KSLWSAYMLMAGVATSRLGLWMFDLAVIQQMQDQVPESDRCVVGGVQ
Ppe2     354 LLLCVASIWVH--NSLLSAYLLMAGVATSRLGLWMFDLAVIQQMQDQVPEFDRCIVGGVQ
Mes2     351 LLLCIGSIWVQ--NRQLSACMLMAGVATSRLGLWMFDLSVIQQMQDQVSESDRCVVGAVQ
Mes3     351 LLLCVASIWVQ--NNLLSAYMLMAGVATSRLGLWMFDLSVIQQMQDHVPESDRCVVGGAQ
Mes4     351 LLLCVASIWVQ--NNLLSAYMLMAGVATSRLGLWMFDLSVIQQMQDHVPESDRCVVGGAQ
Tca2     352 LLLCVASIWVK--NSHLSAYMLMGGVATSRLGLWMFDLSVIQQMQDLVPESN--------
Vvi2     351 LLICIASIWIQ--NHITSAYLLMVGVATSRLGLWMFDLSVIQQMQDHVPESDRCVVGGVQ
Vvi3     343 LLLCVASIWIH--NHIISAYLPMVGVATSRLGLWMFDLSVTQQMQDGVPESDRCVVGGVQ
```

```
AcoMar  382 VSIASLAEVMMLGIAIIANDASHFGFLAMLSLSSVVGAAWMFCRWLVNPTDAQRRLF
VviMar  372 ASVASLAEFVMLGVAIIANDVSHFGFLAMLSLFSAVGAAWMFCRWLVNPTEEQRSLF
BraMar  384 ISVASLAESLMLGVAIAANDASHFGFLAVLSLLSVVAASLIFCRLLRNPTDEQRRLF
EsaMar  384 ISVASLAESLMLGVAIAANDASHFGFLAVLSLLSVVAATLIFCRLLRNPTDEQRRLF
CgrMar  383 ISVASLAESLMLGVAIAANDASHFGFLAVLSLLSVVAASLIFCRLLRNPTDEQRRLF
BstMar  384 ISVASLAESLMLGVAIAANDASHFGFLAVLSLLSVVAASLIFCRLLRNPTDEQRRLF
AthMar  384 ISVASLAESLMLGVAIAANDASHFGFLAVLSLLSVVAASLIFCRLLRNPTDEQRRLF
AlyMar  384 ISVASLAESLMLGVAIAANDASHFGFLAVLSLLSVVAASLIFCRLLRNPTDEQRRLF
CsaMar  382 VSVASLAESIMLGVAIIANDTSHFGFLAMLSLLSVVGAAVIFCQWLLNPTDEQRKLF
FveMar  385 VAVASLAESAMLGVAIIANDVSHFGFLAMLSLLSVIAAAWMFCRWLLNPTDEQRSLF
PpeMar  385 VAVASLAEFTMLGVAIVVNDVSHFGFLAVLSLLSVVGAAWMFCRWLLNPTDEQRTLF
MesMar  385 ISVASLAESLMLGVAIIANDISHFGFLAMLSLLSVVGAAWMFCRWLSNPTDEQRSLF
TcaMar  386 ISVASLAESVMLGVAIIANDVSHFGFLAMLSLLSAVGAAWMFCRWLLNPTEEQRNLF
MguMar  386 VAVASLAESAMLGVAIIANDVSNFGVLAVLSLLSVVGATCLYCSWLKSPTDTQRSLF
SlyMar  383 VSVASLAESIMLGVAIVVNDVSHFGFLATLSLVSVVGAACLYCRWLENPTDTQRTLF
StuMar  383 VSVASLAESIMLGVAIVVNDVSHFGFLATLSLVSVVGAACLYCRWLENPTDTQRTLF
Bra3    406 NSLQSALDLMAYVLGIIVSNPKDFWILTIISFSTVTLAGLIYTVHLYR---IRNHIF
Esa3    405 NSLQSALDLMAYLLGIIVSNPKDFWILTIVSFSTVTLAGLIYTVHLYR---IRNHIF
Ath3    409 NSLQSALDLMAYLLGIIVSNPKDFWILTLISFSTVSLAGMLYTIHLYR---IRNHIF
Aly3    409 NSLQSALDLMAYLLGIIVSNPKDFWILTLISFSTVSLAGMLYTIHLYR---IRNHIF
Bst3    409 NSLQSALDLMAYLLGIIVSNPKDFWILTLISFSTVTLAGMLYTIHLYR---IRNHIF
Cgr3    409 NSLQSALDLMAYLLGVIVSNPKDFWILTLISFSTVTLAGMLYTMHLYR---IRNHIF
Esa2    411 NSLQAALDLMANLLGIIVSDPKDFRILTLFSFSTVSLAGILYTVHVHR---IRKHIF
Bra2    401 NSLQAALDLMANLLGIIVSNPKDFWILTLISFATVSLAGVLYTVHLYR---IRKHLF
Ath2    409 NSLQSALDLMANLLGIIVSNPKDFWMLTLISFATVSLAGILYTIHLYR---IRKHLF
Aly2    409 NSLQAALALMANLLGIIVSNPKDFWMLTLISFATVSFAGMLYTIHLYR---IRKHLF
Bst2    412 NSLQAAFDLMANLLGIIVSNPKDFWMLTLISFSTVSLAGILYTIHLYR---IRKHLF
Cgr2    412 NSLQAALDLMANLLGIIVSNPKDFWMLTLISFGTVSLAGILYTVHLYR---IRKHLF
Aco2    404 HSIQSMMDLLTCIMGIFISNPKDFWKLSVLSFSSVTLAAVLYCFHIYR---VRKHLF
Mgu2    398 SSLQSTLDLMTYIMGIIISNPQEFWKLTMLSFVLVTIAAVLYSVHIYR---VRKHLF
Sly2    408 NSLQSVLELMTYLSGIIISNPQDFWKLILLSFLLVTLASTLYSIHVYR---VRKHLF
Stu2    408 NSLQSFLELMTYLSGIIISNPQDFWKLILLSFLLVTLASTLYSIHVYR---VRKHLF
Csa2    395 NALQSTMDLMGYVMGVIISNPQDFWKLILISFTAVTLAALLYTIHLYR---IRKHLF
Fve2    410 NSLQSIMDLLGYVMGLIISNPQEFWKLTLVSFAVVTLAALLYTLHLYR---IRKHLL
Ppe2    412 NSLQSGMDLMGYVMGIIISNPQDFWKLNLLSFAAVTLAALLYTLHLYR---IRKHLL
Mes2    409 NSLQSILDLLGYVMGIIISHPRDFWKLTLISFSMVTLAALLYSIYLFRW--HRKVLV
Mes3    409 NSLQSTLDLLGYVMGIIISNPQDFWKLILISFSTVTLAALLYSIHVYR---VRKHLF
Mes4    409 NSLQSTLDLLGYVMGIIISNPQDFWKLILISFSTVTLAALLYSIHVYR---VRKHLF
Tca2        ---------------------------------------------------------
Vvi2    409 NSLQSYLDLMAYVMGIIISNPQDFWKLTLLSISAVTLAAVLYSSYIYR---VRKHLF
Vvi3    401 NSLQSYLDLMAYVMGIIISNPQDFWKLTLLSFSAVTVAALLYTLHTYR---VRKHLF
```

Supplementary Figure 2. Multiple alignment of *At*MAR1 orthologs from 16 plant species. The alignment was generated with MUSCLE (https://www.ebi.ac.uk) and shaded with BOXSHADE (https://embnet.vital-it.ch). Phylogenetic analysis was performed using this alignment.

A H387

```
      WT   AATAAACAATGGTGGTTGTCGTTTCTCACGCTCAGCCTTGGTCCGCCTTCCTTACTCTC
Line1 A1   AATAA----------------------------------------------TTACTCTC
      A2   AATAAACAATGGTGGTTGTCGTTTCTCACGCTCAGCCTTGGTCCGCCTTCCTTACTCTC
Line2 A1   AATAAACAATGGTGGTTGTCGTTTCTCACGCTCAG------TCCGCCTTCCTTACTCTC
      A2   AATAAACAATGGTGGTTGTCGTTTCTCACGCTCAGCCTTGGT-----------------
Line3 A1   AATAAACAATGGTGGTTGTCGTTTCTCACGCTCAGCCTTGGTTCCGCCTTCCTTACTCTC
      A2   AATAAACAATGGTGGTTGTCGTTTCTCACGCTCAGCCTTGGTCCGCCTTCCTTACTCTC

      WT   CGTCAAGTTCCTCTTACCTCCTCCTCTTCTTTTAGAATACGAACCTTAACTCCTCGCTT
Line1 A1   CGTCAAGTTCCTCTTACCTCCTCCTCTTCTTTTAGAATACGAACCTTAACTCCTCGCTT
      A2   CGTCAAGTTCCTCTTACCTCCTCCTCTTCTTTTAGAATACGAACCTTAACTCCTCGCTT
Line2 A1   CGTCAAGTTCCTCTTACCTCCTCCTCTTCTTTTAGAATACGAACCTTAACTCCTCGCTT
      A2   -----------------------------------------------------------
Line3 A1   CGTCAAGTTCCTCTTACCTCCTCCTCTTCTTTTAGAATACGAACCTTAACTCCTCGCTT
      A2   CGTCAAGTTCCTCTTACCTCCTCCTCTTCTTTTAGAATACGAACCTTAACTCCTCGCTT

      WT   TCGATCGTTAACTCCTCGCTTTACCACGCAAAGGTGCGATGCTTTTCCTTGTAAATTGA
Line1 A1   TCGATCGTTAACTCCTCGCTTTACCACGCAAAGGTGCGATGCTTTTCCTTGTAAATTGA
      A2   TC---------------------------------------------------------
Line2 A1   TCGATCGTTAACTCCTCGCTTTACCACGACAAAGGTGCGATGCTTTTCCTTGTAAATTGA
      A2   -----------------------------AAAGGTGCGATGCTTTTCCTTGTAAATTGA
Line3 A1   TCGATCGTTAACTCCTCGCTTTACCAC-CAAAGGTGCGATGCTTTTCCTTGTAAATTGA
      A2   TCGATCGTTAACTCCTCGCTTTACCAC-CAAAGGTGCGATGCTTTTCCTTGTAAATTGA

      WT   CAATGTAAATGAATGAGTTGCTACTGTGTGATCATGATCTTAC
Line1 A1   CAATGTAAATGAATGAGTTGCTACTGTGTGATCATGATCTTAC -46 bp
      A2   -----------------------------GATCATGATCTTAC -86 bp
Line2 A1   CAATGTAAATGAATGAGTTGCTACTGTGTGATCATGATCTTAC -6 bp, +1 bp
      A2   CAATGTAAATGAATGAGTTGCTACTGTGTGATCATGATCTTAC -112 bp
Line3 A1   CAATGTAAATGAATGAGTTGCTACTGTGTGATCATGATCTTAC +1 bp, -1 bp
      A2   CAATGTAAATGAATGAGTTGCTACTGTGTGATCATGATCTTAC -1 bp
```

B H386

```
      WT  AGATTTCTGATGTCAAGTGCATTCAAACAGCTTTATATGCTAGTTGCTTGGCTGGGTAT
Line4 A1  AG---------------------------------------------------------
      A2  AGATTTCTGATGTCAAGTGCATTCAAACAGCTTTAT-----------------------
      A3  AGATTTCTGATGTCAAGTGCATTCAAACAGCTTTATATGCTAGTTGCTTGGCTGGGTAT
Line5 A1  AGATTTCTGATGTCAAGTGCATTCAAACAGCTTTATATGCT------------------
      A2  AGATTTCTGATGTCAAGTGCATTCAAACAGCTTTATATGCTAGTTGCTTGGCTGGGTAT
Line6 A1  AGATTTCTGATGTCAAGTGCATTCAAACAGCTTTATATGCTAGTTGCTTGGCTGGGTAT
      A2  AGATTTCTGATGTCAAGTGCATTCAAACAGCTTTATATGCTAGTTGCTTGGCTGGGTAT
Line7 A1  AGATTTCTGATGTCAAGTGCATTCAAACAGCTTTAT-----------------------
      A2  AGATTTCTGATGTCAAGTGCATTCAAACAGCTTTATATGCTAGTTGCTTG---------
      A3  AGATTTCTGATGTCAAGTGCATTCAAACAGCTTTATATGCTAGTTGCTTGGCTGG--AT

      WT  ATGGTGGAACAACTTTGGAATTTCGCCTCACCTGCTGCCATTGCGTTGATT
Line4 A1  ---------------------------------------------TTGATT -102 bp
      A2  ---------------------TTCGCCTCA----CTGCCATTGCGTTGATT -44 bp, -5 bp
      A3  --GGTGGAACAACTTTGGAATTTCGCCTCAC-TGCTGCCATTGCGTTGATT -2 bp, -1 bp
Line5 A1  ------------------------------------GCCATTGCGTTGATT -54 bp
      A2  --GGTGGAACAACTTTGGAATTTCGCCTCACCTGCTGCCATTGCGTTGATT -2 bp
Line6 A1  ----------AACTTTGGAATTTCGCCTCACCTGCTGCCATTGCGTTGATT -10 bp
      A2  --GGTGGAACAACTTTGGAATTTCGCCTCACCTGCTGCCATTGCGTTGATT -2 bp
Line7 A1  ---------------------TTCGCCTCACCTGCTGCCATTGCGTTGATT -44 bp
      A2  ----------------------------------CTGCCATTGCGTTGATT -43 bp
      A3  ATGGTGGAACAACTTTGGAATTTCGCCTCACCTGCTGCCATTGCGTTGATT -2 bp
```

Supplementary Figure 3. Mutated *SlMAR1* alleles detected in regenerated shoots selected on kanamycin-containing medium. (A) Sequences from shoots transformed with H387. (B) Sequences from shoots transformed with H386. The PAM sequence is indicated with bold letters and the gRNA target sites are highlighted in grey.
